# Development of Biomarkers for Screening Hepatocellular Carcinoma Using Global Data Mining and Multiple Reaction Monitoring

**DOI:** 10.1371/journal.pone.0063468

**Published:** 2013-05-22

**Authors:** Hyunsoo Kim, Kyunggon Kim, Su Jong Yu, Eun Sun Jang, Jiyoung Yu, Geunhee Cho, Jung-Hwan Yoon, Youngsoo Kim

**Affiliations:** 1 Department of Biomedical Engineering, Seoul National University College of Medicine, Seoul, Republic of Korea; 2 Department of Internal Medicine, Seoul National University College of Medicine, Seoul, Republic of Korea; 3 Departments of Life Science and Biotechnology, Yonsei University, Seoul, Republic of Korea; National Cancer Institute, United States of America

## Abstract

Hepatocellular carcinoma (HCC) is one of the most common and aggressive cancers and is associated with a poor survival rate. Clinically, the level of alpha-fetoprotein (AFP) has been used as a biomarker for the diagnosis of HCC. The discovery of useful biomarkers for HCC, focused solely on the proteome, has been difficult; thus, wide-ranging global data mining of genomic and proteomic databases from previous reports would be valuable in screening biomarker candidates. Further, multiple reaction monitoring (MRM), based on triple quadrupole mass spectrometry, has been effective with regard to high-throughput verification, complementing antibody-based verification pipelines. In this study, global data mining was performed using 5 types of HCC data to screen for candidate biomarker proteins: cDNA microarray, copy number variation, somatic mutation, epigenetic, and quantitative proteomics data. Next, we applied MRM to verify HCC candidate biomarkers in individual serum samples from 3 groups: a healthy control group, patients who have been diagnosed with HCC (Before HCC treatment group), and HCC patients who underwent locoregional therapy (After HCC treatment group). After determining the relative quantities of the candidate proteins by MRM, we compared their expression levels between the 3 groups, identifying 4 potential biomarkers: the actin-binding protein anillin (ANLN), filamin-B (FLNB), complementary C4-A (C4A), and AFP. The combination of 2 markers (ANLN, FLNB) improved the discrimination of the before HCC treatment group from the healthy control group compared with AFP. We conclude that the combination of global data mining and MRM verification enhances the screening and verification of potential HCC biomarkers. This efficacious integrative strategy is applicable to the development of markers for cancer and other diseases.

## Introduction

Hepatocellular carcinoma (HCC) is the fifth most common cancer worldwide and the third leading cancer-related cause of death [1]. Since many HCCs are asymptomatic before the development of end stage disease, regular surveillance for HCC is mandatory for patients with chronic hepatitis or cirrhosis to detect a tumor at an early stage and to improve patients’ outcomes after curative treatment [2]. Currently, most practice guidelines recommend routine surveillance for HCC using ultrasonography and serum tumor markers, such as alpha-fetoprotein (AFP). [3], [4], [5] However, the use of AFP as a single biomarker for HCC is challenging due to its limited specificity and sensitivity.

Tumor biomarkers are defined as substances that reflect current cancer status or predict its future characteristics. Biomarkers are potentially useful for screening cancers and determining their prognosis, predicting therapeutic efficacy [6]. The most commonly used serum marker of HCC is AFP, which has a reported sensitivity of 39% to 65% and specificity of 65% to 94%; approximately one-third of early-stage HCC patients with small tumors (<3 cm) have normal levels of AFP [2], [7]. Thus, clinicians are dissatisfied with AFP as a marker due to its high false-positive and false-negative rates [8]. Consequently, there is an urgent clinical need to identify new biomarkers that classify HCC more accurately.

To obtain HCC biomarker candidates, we initially screened a published database on HCC using 5 types of datasets, comprising proteomics, cDNA microarray, copy number variation, somatic mutation, and epigenetic data. This method easily encompassed all biological heterogeneities of liver cancer. The candidates that resulted from global data mining were subject to high-throughput verification using individual HCC serum samples by multiple reaction monitoring (MRM) [9]. In MRM verification, specific peptides of candidates are selected to represent the protein from which they are quantitated against a spiked internal standard (a synthetic stable isotope-labeled peptide), yielding a measure of its concentration [10].

Three clinically well-characterized serum samples–from the healthy control, Before HCC treatment, and After HCC treatment groups–were used to quantify the candidate biomarkers, of which we identified significant candidates for differentiation between the before the former and latter groups. Two MRM-verified biomarkers were distinguished between the 3 groups. Further, in combination, this 2-marker panel distinguished the groups better than the individual markers.

In this study, MRM verification was combined with global data mining to verify the biomarker candidates that were screened from an initial global data mining step in identifying and developing valuable HCC biomarkers. The MRM verification resulted in 9 potential markers with an area under the curve (AUC) that exceeded 0.7, wherein 2 of the 9 verified markers were combined to construct a 2-marker panel by multivariate analysis. The 2-marker panel had an improved AUC compared with AFP (0.981 versus 0.756, respectively). This approach enabled us to verify HCC biomarkers–especially a promising multimarker panel that can be used to improve HCC detection alone or in combination with AFP levels.

## Materials and Methods

### Ethics Statement and Clinical Sample Information

The institutional review board of Seoul National University Hospital (approval No. H-1103-056-355) approved the study protocol, and written informed consent was obtained from each patient or legally authorized representative. The clinical characteristics of the study patients are shown in Table 1.

**Table 1:** Clinical characteristics of patient groups used in MRM analysis and Western blot analysis.

	MRM analysis		Western blot analysis	
	Before HCC treatment groupand corresponding afterHCC treatment group	Healthycontrol group	Before HCC treatment groupand corresponding afterHCC treatment group	Healthy control group
**Total patient number**	18 in each group	36	13 in each group	13
**Gender (Male/Female)**	13/5	18/18	10/3	8/5
**Age (Mean, Range)**	60.6 (47–81)	58.7 (50–69)	62.4 (48–79)	56.2 (52–67)
**Etiology of liver disease**	HBV, 18 (100%)		HBV, 13 (100%)	
**Locoregional modality**				
TACE	7		4	
PEIT	11		9	
**AFP value** **(Mean, Range)**	1079.4 (14.1–6900)		245.2 (16–730)	
<20 ng/mL	2		1	
20–200 ng/mL	4		6	
200–1000 ng/mL	7		6	
>1000 ng/mL	5		0	
**PIVKA value** **(Mean, Range)** *	916 (5–10720)		117.6 (28–612)	
<20 ng/mL	4		0	
20–100 ng/mL	6		7	
100–1000 ng/mL	3		6	
>1000 ng/mL	3		0	

* PIVKA values were provided for 16(M11/F5) among a total of 18 untreated HCC group.

AFP : Alpha-Fetoprotein.

PIVKA : Protein induced by vitamin K absence or antagonist.

TACE : Transcatheter arterial chemoembolization.

PEIT : Percutaneous ethanol injection therapy.

Healthy control group samples were obtained from 36 healthy volunteers who visited the Healthcare Center of Seoul National University Hospital. All subjects in healthy control group were confirmed with normal liver function test results, including serum alanine and aspartate aminotransferases, and with negative results for hepatitis B virus surface antigen and anti-hepatitis C virus antibody. Liver ultrasonography was performed to screen fatty liver disease, and all healthy controls had normal findings. Eighteen patients before HCC treatment who were infected with hepatitis B virus (HBV) and underwent successful locoregional therapy were also enrolled, from whom serum samples were collected and classified as the Before and After HCC treatment groups, respectively.

The diagnosis of HCC was based on the recommendation of the American Association for the Study of Liver Diseases by a hepatologist with more than 20 years of experience [11]. All HCC patients were after treatment with locoregional modality including transarterial chemoembolization and percutaneous ethanol injection therapy. The treatment response was evaluated with serum AFP and enhanced liver computed tomography (CT) at 3 months after the first treatment, and no enrolled patient showed any evidence of tumor recurrence. In each HCC patient, serum samples were obtained twice: before the first locoregional therapy (Before HCC treatment group), and at 3 months after the treatment (After HCC treatment group) (Supplementary Table S1). To reduce causal heterogeneity, HCC patients who had other types of chronic liver disease, except chronic hepatitis B, such as chronic hepatitis C and alcoholic hepatitis, were excluded.

All serum samples were collected by the Liver Research Institute, Seoul National University College of Medicine. The blood samples were centrifuged immediately at 3000 rpm for 10 min at 4°C to fractionate the serum. The resulting supernatant was aliquoted (50 µL) and stored at −80°C until analysis.

### Preparation of Serum Tryptic Digestions

Serum protein was quantified by bicinchoninic acid (BCA) assay. Two hundred-microgram aliquots of the serum samples were denatured with 6 M urea, 50 mM Tris, pH 8.0, and 30 mM dithiothreitol (DTT) at 37°C for 60 min and alkylated with 50 mM iodoacetamide (IAA) at room temperature in the dark for 30 min. The urea was diluted 15-fold with 50 mM Tris, pH 8.0 prior to overnight digestion at 37°C with trypsin (Promega, sequencing-grade modified) using a 1∶50 (w/w) enzyme-to-serum concentration ratio.

Tryptic digestion was stopped with formic acid at a final concentration of 1% and desalted on Sep-pak tC18 cartridges (Waters Corp., Milford, MA). The Sep-pak tC18 cartridges were equilibrated sequentially with 1 mL methanol and 5 mL water that contained 0.1% trifluoroacetic acid (TFA) prior to loading of the tryptic digestion. The cartridges were washed with 3 mL 0.1% trifluoroacetic acid (TFA) and eluted with 1 mL of 60% ACN, 0.1% TFA. The eluted samples were frozen and lyophilized on a speed vacuum. Prior to MRM analysis, the samples were reconstituted in 0.1% formic acid to 2 µg/µL.

### Experimental MRM Design Using Skyline

For each target protein, we selected peptides and fragment ions for MRM using Skyline (http://proteome.gs.washington.edu/software/skyline), an open-source software application for developing MRM methods and analyzing MRM data [12]. In brief, the full-length protein sequences were imported into Skyline in FASTA format and designed into peptides, each with a list of product ions for monitoring by MRM. In selecting transitions through Skyline, the peptide filter condition was as follows: maximum length of peptide of 20, including at least 8 amino acids. Peptides with repeat arginines (Arg, R) or lysines (Lys, K) were discarded. If methionine (Met, M) was included in the peptide, it was discarded to avoid the risk of modification. If proline (Pro) lay next to arginine (Arg, R) or lysine (Lys, K), the peptide was discarded. If a peptide contained histidine (His, H), it was discarded to avoid the risk of charge alteration. Peptides that satisfied these conditions were used as Q1 transitions. Next, we selected a maximum of 5 Q3 transitions from the fragmentation ions that were derived from the Q1 transitions in descending order.

### Quantification by Multiple Reaction Monitoring

MRM was performed on a nano LC system, which was connected to a hybrid triple quadrupole/ion trap mass spectrometer (4000 QTRAP, AB SCIEX, Foster City, CA) that was equipped with a nanoelectrospray interface. The 4000 QTRAP was operated in positive ion MRM mode, in which Q1 and Q3 were set to transmit different precursor/product ion pairs.

The LC buffer system was as follows: mobile phase A, 2% acetonitrile/0.1% formic acid and mobile phase B, 98% acetonitrile/0.1% formic acid. The peptides were separated and eluted at a flow rate of 300 nL/min on a linear gradient of mobile phase B from 2% to 40% B in 43 min. The gradient was ramped up to 70% B for 5 min and 2% B for 10 min to equilibrate the column for the next run. The total LC run time was 60 min. The analytical column was 75 µm, 15 cm, packed with Magic C18AQ resin (5 µm, 100 Å, Michrom Bioresources).

Typical instrument settings were as follows: ion spray (IS) voltage of 2.3 kV, an interface heater temperature of 200°C, a GS1 (nebulizer gas) setting of 12, and curtain gas set to 15. MS parameters for declustering potential (DP) and collision energy (CE) were determined by linear regression of previously optimized values in Skyline. MRM experiments were performed with a scan time of 50 ms and scan width of 0.002 m/z, using a unit resolution of 0.7 Da (FWHM) for Q1 and Q3. In the MRM runs, scan time was maintained at 50 ms for each transition, and the pause between transition scans was set to 3 ms [13].

### Statistical Analysis for Verification of Biomarker Candidates

Raw data files from the MRM analysis were processed using Skyline. Because the peak intensity is sometimes low due to low abundance in a normal versus cancer sample and vice versa, the peak area integration was confirmed manually to correct the wrong automatic assignments for each targeted peptide. The default peak integration and Savitzky-Golay smoothing algorithm were applied. Peptides with at least 3-fold signal-to-noise ratios were considered detectable.

Two approaches were used to assess HCC candidate proteins. First, we distinguished a disease group (Before HCC treatment) from a nondisease group (healthy controls and after HCC treatment). Comparisons between the before HCC treatment (n = 18) versus healthy control groups (n = 36) and the before HCC treatment (n = 18) versus after HCC treatment groups (n = 18) were made using analysis of variance (ANOVA). Based on ANOVA, we selected target peptides that had a significance level below 0.05 in mean intensity level between groups.

Second, to evaluate the efficacy of serum biomarkers in distinguishing the disease from nondisease group, we analyzed receiver operator characteristic (ROC) curves and scatter plots. We performed all statistical analyses and generated all scatter plots and ROC curves with MedCalc (MedCalc, Mariakerke, Belgium, version 12.2.1).

### Western Blot Analysis

The clinical samples in the Western blot experiment comprised 13 individual samples from the healthy control, before HCC treatment, and after HCC treatment groups (Table 1). Serum sample concentrations were determined by BCA protein assay. Equal amounts of protein (30 µg) were mixed with SDS loading buffer (62 mM Tris-HCl, pH 6.8, 10% glycerol, 2% SDS, 2% ß-mercaptoethanol, and bromophenol blue), boiled for 10 min, and separated by SDS-PAGE on a 12% acrylamide gel. After separation, serum samples were transferred to polyvinylidene difluoride (PVDF) membranes (Bio-Rad, Cat. #162-0177), which were blocked with 5% BSA (w/v) in TBS-T (25 mM Tris, pH 7.5, 150 mM NaCl, and 0.05% (w/v) Tween-20) for 2 hr at room temperature.

Membranes were incubated overnight at 4°C with individual primary antibodies (diluted 1∶100 to 1∶1000). Membranes were washed 5 times with TBS-T and incubated for 2 hr with the appropriate secondary peroxidase-conjugated antibody (1∶5000, Santa Cruz Biotechnology, USA). The membranes were then washed 5 times with TBS-T, and target protein bands were visualized using the Chemiluminescent Substrate Kit (GenDEPOT, W3651-012). Western blot band intensities were quantified using Multi Gauge. (Fujifilm, ScienceLab 2005, version 3.0). Pooled serum was used as a loading control (13 healthy control, 13 untreated HCC, and 13 treated HCC group samples) and in each Western blot gel. All blots were normalized to the band intensity of the pooled serum. Band intensities were analyzed by *T*-test to identify meaningful differences between sample groups.

### Statistical Analysis to Construct Multimarker Panel

In this study, we compared the nondisease with the disease group using multimarker panel proteins. Logistic regression (LR) analysis was performed to generate the multimarker panel, consisting of several individual markers that differentiated cancerous from noncancerous subjects. The discriminatory power was examined in 3 situations: healthy control versus before HCC treatment groups, before HCC treatment versus after HCC treatment groups, and healthy control plus after HCC treatment versus before HCC treatment groups. LR and ROC curves were constructed using MedCalc, and the analysis was performed.

ROC curves were used to evaluate the efficacy of the multimarker panel–in this case, a 2-marker panel. AUC values for individual markers and the 2-marker panel were calculated to examine the discriminatory power of various combinations of HCC candidate markers. Multicolinearity of the panel was checked using IBM SPSS Statistics (version 20, commuter license).

Leave-one-out crossvalidation (LOOCV) was performed to avoid overfitting that might be caused by the small number of samples, even if LOOCV is a suboptimal substitution of independent validation; thus, the same sample set was used to generate the training and test data. A single observation was selected from the dataset as the test variable, and the remaining samples were used as the training set to construct an LR model. LOOCV was performed in the open-source Weka program computing environment (version 3.6.0, Knighton Rd, Hamilton 3240 New Zealand).

## Results

### Candidate Biomarker Selection from Global Data Mining

**Figure 1:**
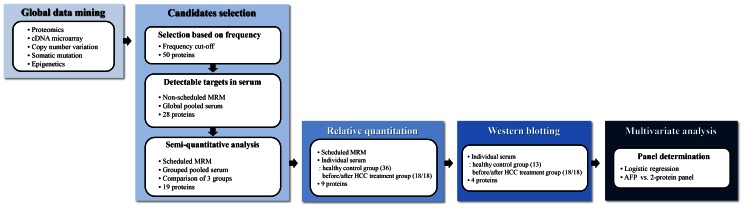
Workflow of HCC biomarker discovery. First, we selected candidate HCC biomarkers, based on global data mining using preexisting databases. In the first and second screening steps, preliminary MRM analysis of the target peptides/transitions was conducted using pooled serum samples to examine whether the transitions were detectable in serum samples. In the first verification step, MRM analysis of individual serum samples was performed using the predetermined retention time. In the second verification step, the data were analyzed and verified by Western blot. In the multivariate analysis, logistic regression (LR) analysis was performed to construct a multimarker panel of potential markers that could differentiate cancerous from noncancerous subjects.

The overall scheme for this study is shown in Figure 1. Our first task was to obtain a list of biomarker candidate proteins. We believed that if we could acquire candidate proteins from established resources, the number of candidate screening experiments could be reduced. To screen candidates, we performed global data mining with regard to liver cancer in several disciplines: proteomics, cDNA microarray, copy number variation, epigenetics, and somatic mutation data.

The second task was to prioritize marker candidates from the resulting list (Supplementary Table S2). The term “frequency” was used for each target protein. Frequency was defined as the total number of occurrences in 5 biological fields. As a result of data mining of these 5 areas, 4658 liver cancer-related proteins were selected and filtered by prioritizing candidate proteins. The top 50 high-frequency proteins were chosen and examined with regard to whether they were secreted into plasma, and final candidates were selected by confirmation with the Plasma Proteome Database (http://www.plasmaproteomedatabase.org). All 4658 proteins from the global data mining are listed in Supplementary Table S2. Then, sequence files of the 50 selected candidates were prepared in FASTA format and harvested using the Uniprot website (http://www.uniprot.org). The 50 FASTA files were input into Skyline to generate theoretical transitions for the MRM analysis.

The list of potential biomarkers was filtered per the verification steps, as summarized in the summary list file (Supplementary Table S3).

### Data Mining of Proteomic Research

Ten research papers, all published after 2004, were selected with impact factors above 4.0 [14], [15], [16], [17], [18], [19], [20], [21], [22], [23]. Two ICAT labeling, 4 ITRAQ labeling, and 4 SILAC labeling reports were used for our proteomic data mining. The maximum frequency of the target proteins in the 10 journals was 5. Based on the frequency, the most commonly reported genes were vimentin (VIM), catechol O-methyltransgerase (COMT), enoyl-CoA hydratase, mitochondrial (ECHS1), and transitional endoplasmic reticulum ATPase (VCP), which were reported 5 times.

### Data Mining of cDNA Microarray Research

cDNA microarray research papers that examined gene expression using liver cancer and control tissues were examined. Nine such reports were selected (all published after 2003), with impact factors above 6.7 [24], [25], [26], [27], [28], [29], [30], [31], [32]. The total number of screened proteins was 3241, and the most cited gene was liver carboxylesterase 1 (CES1), which was reported in 6 papers.

### Data Mining of Copy Number Variation Research

Earlier publications on copy number variation (CNV) by amplification or mutation of liver cancer [33], [34], [35] were investigated, yielding 3 papers after 2004 with impact factors above 4.4. OncoDB (http://oncodb.hcc.ibms.sinica.edu.tw/index.htm) was also used to report copy number variation. In the 3 papers and OncoDB, CNVs in exostosin-1 (EXT1), transforming growth factor beta-2 (TGFB2), RAC-gamma serine/threonine-protein kinase (AKT3), and cathepsin B (CTSB) were reported twice.

### Data Mining of Epigenetic Research

On surveying epigenetic research papers, we selected 3 studies from after 2005 with impact factors above 4.3 [28], [36], [37], all of which reported cyclin-dependent kinase inhibitor 2A, isoform 4 (CDKN2A).

### Data Mining of Somatic Mutation Research

Three databases on somatic mutations were searched: the OncoDB (http://oncodb.hcc.ibms.sinica.edu.tw/index.htm), Japan Liver Cancer (NCC, Riken), and International Cancer Genome Consortium (http://www.icgc.org/icgc/cgp/66/420/824) databases. Using HCC as the query term, 102 proteins (54, 25, and 23 proteins in NCC, Riken, and Onco.DB.HCC, respectively) were screened for somatic mutation data.

### Examine the Linearity of the Internal Standard Peptide

**Figure 2:**
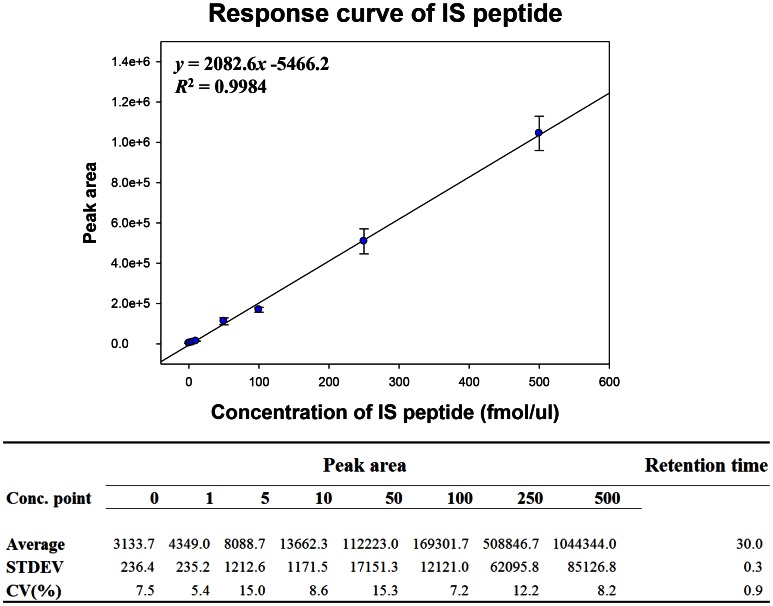
Response curve using ATP-dependent RNA helicase A (DHX9) peptide. MRM runs were performed using an internal standard peptide (ELDALDANDELTPLGR) of ATP-dependent RNA helicase A (DHX9) at a Q1/Q3 transition of 876.4/1095.57 m/z, with which the standard curve was drawn. Triplicate MRM analyses were performed at 8 concentrations of the peptide (0, 1, 5, 10, 50, 100, 250, 500 fmol). The curve showed a linearity of *R^2^* = 0.9984.

The peak area of the internal standard peptide in each sample was used to normalize that of each target peptide in each MRM run. Thus, to assess the quantitative linearity of MRM, a response curve of the standard peptide (sequence of ELDALDANDELTPLG***R***, ATP-dependent RNA helicase A (DHX9)) in the presence of matrix peptides was generated. The internal standard peptide was diluted serially to 1, 5, 10, 50, 100, 250, and 500 fmol in 200 µg of serum peptide mixture, which is similar to the MRM conditions for target peptides. Further, to verify the endogenous signal of the target peptide, a blank sample that lacked the internal standard peptide was run. Each MRM analysis was performed three times repeatedly (Fig. 2).

The CV% of the experiment was below 20%, and the correlation coefficient was 0.9984. The response curve of the standard peptide with the serum peptide mixture as matrix indicated that the quantitative linearity of serum MRM at the given concentrations was sufficiently valid to obtain relative quantities of target peptides.

### Detectability of Target Candidates in Pooled Serum

Before conducting individual MRM analyses using the 72 serum samples, a preliminary MRM analysis was performed on the target peptides/transitions using pooled serum of all patients to obtain transition information, such as the detectability in serum and the suitability of the transition. The FASTA files of the 50 proteins yielded 498 peptides and 2174 transitions on applying the hierarchy data in Skyline software. After obtaining the resulting MRM data, the final transition was selected using the following criteria: at least 2 peptides were selected per protein, and at least 3 transitions per peptide were chosen as detectable transitions that had a signal-to-noise (S/N) ratio above 3. Ultimately, 28 of 50 candidates met the criteria in the first screening step (Supplementary Table S3).

### Selecting the Transitions with Technical Reproducibility for MRM Analysis

The reproducibility of MRM analyses is critical in making quantitative measurements [9]. In this study, the technical reproducibility of MRM analysis was examined using pooled serum. Serum from the 3 groups (36 healthy control, 18 before and after HCC treatment each) was pooled with the same weight according to each group; 333 transitions for 111 peptides, corresponding to the 28 candidate proteins from the detectability experiment (the first screening step, Supplementary Table S3), were used to determine the technical reproducibility in MRM analysis. Five repetitive scheduled MRM runs for the pooled serum peptide mixture were performed using the retention time from the detectability experiment, with a window size of 120 seconds.

The data from the 5 MRM runs were imported into Skyline, and after normalization of the peak area using the internal standard peptides, the averaged relative quantities of each transition were compared. Peptides that showed a confident difference in quantity (fold-change >1.5) and low variance (CV <30%) between nondisease and disease groups were chosen as the final quantifiable transitions. Consequently, 30 peptides, comprising 90 transitions that corresponded to 19 proteins, were selected (second screening step, Supplementary Table S3). The average relative quantities and standard deviations from the 5 repeat MRM analyses of the 30 peptides are summarized in Supplementary Table S4, and the final transition list is shown in Supplementary Table S5.

### MRM Analysis Using Individual Serum Samples

Individual MRM analysis was performed using the 90 transitions, corresponding to 19 proteins; thus, MRM analysis per run was conducted once for every sample. The peak areas of each transition were extracted using Skyline and normalized using the peak area of the spiked standard peptide.

Nine proteins had identical expression patterns in the analysis of the pooled and individual samples (Supplementary Figure S1). Specifically, very-long-chain-specific acyl-coA dehydrogenase (ACADVL), actin-binding protein, anillin (ANLN), c-1-tetrahydrofolate synthase, cytoplasmic (MTHFD1), alpha-fetoprotein (AFP), and filamin-B (FLNB) increased in the healthy control versus before HCC treatment group and declined in the before HCC treatment versus after HCC treatment group. Brain acid-soluble protein 1 (BASP1), calpain-1 catalytic subunit (CAPN1), complementary C4-A (C4A), and polyadenylate-binding protein 1 (PABPC1) fell in the healthy control versus the before HCC treatment group and in the before HCC treatment versus after HCC treatment group.

In the statistical analysis, these 9 proteins were differentially expressed between the nondisease and disease groups, with *P*-values <0.05. The ROCs and interactive plots of the 9 candidates are shown in Supplementary Figure S2. The AUC values of the 9 target proteins ranged from 0.586 to 0.951 (Table 2). The AUC values of 6 proteins exceeded 0.8–those of ANLN, BASP1, CAPN1 and PABPC1 were 0.920, 0.951, 0.946 and 0.949, respectively, in the healthy control versus before HCC treatment group, reflecting excellent specificity and sensitivity.

**Table 2:** List of proteins showing significant differences between different groups (*P*-values <0.05) and their respective AUC values.

SerialNo.	Gene Symbol	Protein Name	Peptide Sequence	Area Under ROC curve	
				Healthy control groupvs. Before HCC treatmentgroup	Before HCC treatment group vs. After HCC treatment group
1	ACADVL	Very long-chain specific acyl-CoA dehydrogenase, mitochondrial	ASNTAEVFFDGVR	0.701	0.691
2	AFP	Alpha-fetoprotein	GYQELLEK	0.756	0.735
3	ANLN	Actin-binding protein anillin	TQSLPVTEK	0.920	0.744
4	BASP1	Brain acid soluble protein 1	AEGAATEEEGTPK	0.951	0.719
5	C4A	Complement C4-A	VGDTLNLNLR	0.867	0.710
6	CAPN1	Calpain-1 catalytic subunit	YLGQDYEQLR	0.946	0.586
7	FLNB	Filamin-B	APLNVQFNSPLPGDAVK	0.660	0.707
8	MTHFD1	C-1-tetrahydrofolatesynthase, cytoplasmic	GVPTGFILPIR	0.829	0.685
9	PABPC1	Polyadenylate-binding protein 1	EFSPFGTITSAK	0.949	0.608

### Correlation of MRM Analysis with Western Blot

To validate the MRM results, 9 proteins were analyzed by western blot: ACADVL, AFP, ANLN, BASP1, C4A, CAPN1, FLNB, MTHFD1, and PABPC1.

For the Western blot, 13 of 36 healthy control serum samples from the individual MRM analysis were selected randomly, and the before/after HCC treatment group, the 13 serum samples differed from what was used in the individual MRM analysis.

To limit the variability between SDS-PAGE gels, serum from the 13 healthy control, 13 before HCC treatment, and 13 after HCC treatment cases were pooled and loaded onto the first lane of all SDS-PAGE gels (12%), and its intensity on each gel was used to normalize the intensities of individual samples. As a result, the bands that were generated by ACADVL and PABPC1 were inadequate to calculate intensities; thus, 7 of the 9 candidate proteins were verified by western blot. Subsequently, the correlation between the protein quantification by MRM and western blot was determined.

**Figure 3:**
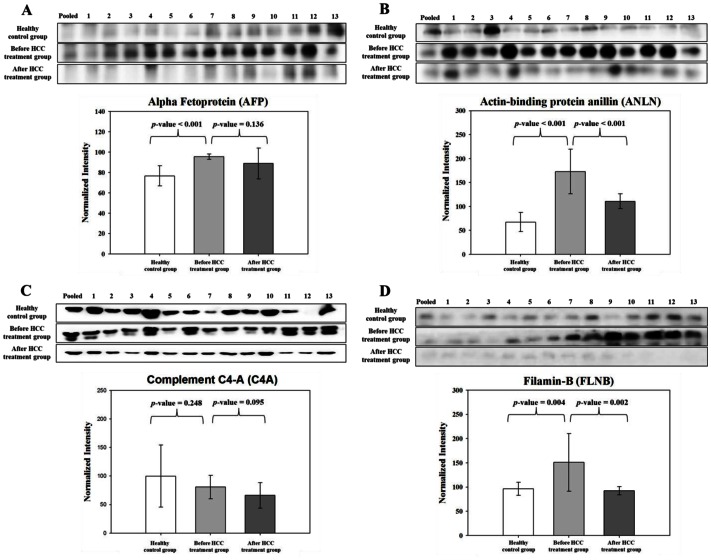
Verification of four proteins by Western blot. Comparison and relative quantification of AFP, ANLN, C4A, and FLNB expression in serum samples from the nondisease group and disease group. Relative abundance, represented by the band intensity in Western blot, is summarized in box plot.

AFP, ANLN, C4A, and FLNB had the same patterns of expression by MRM analysis (Figure 3). In the nondisease group, AFP, ANLN, and FLNB expression decreased compared with disease group. AFP is an established serum biomarker for HCC [38] and was verified to have consistent expression by MRM and Western blot. C4A declined in the healthy control versus before HCC treatment group and in the before HCC treatment versus after HCC treatment group. In the comparison between MRM- and antibody-based verification, the relative quantities of 4 proteins (AFP, ANLN, FLNB, and C4A) were similar. In contrast, by western blot, BASP1, MTHFD1, and CAPN1 had disparate expression patterns compared with MRM analysis (data not shown).

### Multivariate Analysis of the Multimarker Panel

**Figure 4:**
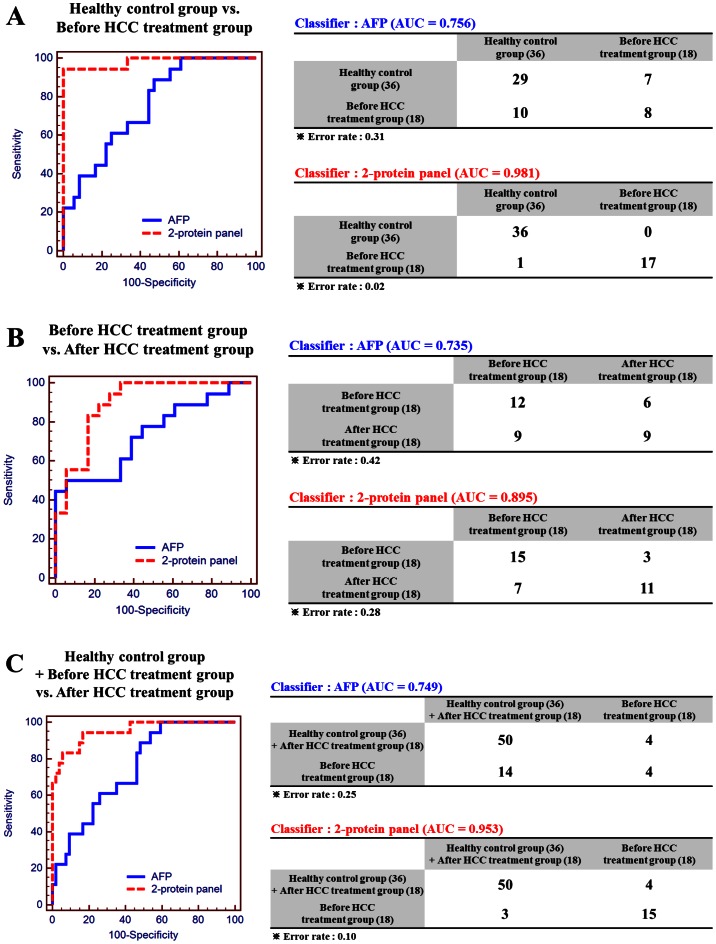
ROC curves of AFP and 2-marker panel. ROC curves are based on prediction models using AFP alone or the 2-marker panel. Simple ROC analysis was performed to compare AFP with the 2-marker panel. LR analysis models were prepared to determine false positive/negative rate in the classifier tables, in which error rates are also shown. Three LR analysis models were prepared: (A) healthy control versus before HCC treatment group, (B) before HCC treatment versus after HCC treatment group, and (C) healthy control plus after HCC treatment versus before HCC treatment group.

A goal of this study was to discover potential HCC biomarkers by comprehensive global data mining and MRM and construct a multiprotein panel has improved discriminatory power over single markers. Four proteins that were validated by MRM analysis and western blot were used to generate the multimarker panel. In the first 4-marker panel that we attempted, AFP and C4A showed collinearity, with a variance inflation factor (VIF) above 10; thus, these proteins were excluded in the final multimarker model (Supplementary Table S6). The ultimate model comprised ANLN and FLNB, for which AFP was the control model as a single marker. Three types of comparisons were made (Figure 4).

When AFP was used as the lone classifier, 8 of 18 before HCC treatment cases and 29 of 36 healthy control cases were classified correctly, demonstrating an AUC of 0.756 and 31% error rate. In contrast, the 2-marker panel correctly classified 17 of 18 before HCC treatment patients and all 36 healthy controls, with an AUC of 0.981 and 2% error rate, indicating that the 2-marker panel had improved discriminatory power compared with the AFP model.

The AFP-only model classified 12 of 18 before HCC treatment patients and 9 of 18 after HCC treatment patients correctly (AUC = 0.735, error rate = 42%), whereas the 2-marker panel separated 15 of 18 before HCC treatment and 11 of 18 after HCC treatment patients successfully (AUC = 0.895, error rate = 28%). Thus, in identifying patients in the before HCC treatment versus after HCC treatment groups, the discriminatory power of the 2-marker panel was outstanding compared with AFP alone.

The AFP-only model classified 4 of 18 in the disease group (before HCC treatment) and 50 of 54 in the nondisease group (healthy control and after HCC treatment groups), with an AUC of 0.749 and error rate of 25%. The 2-marker panel identified 15 of 18 in the disease group and 50 of 54 in the nondisease group (AUC = 0.953, error rate = 10%). These results demonstrate that in all comparisons, the 2-marker panel has greater discriminatory power compared with the traditional single marker AFP.

Supplementary Table S7 also shows the LOOCV results for AFP and the 2-protein panel. LOOCV, in which each member of the training set, using a model that was built with the other n–1 members, one tries to predict the class of the remaining member–was also performed. The results indicate that the most accurate candidate biomarker in the respective groups was the 2-protein panel.

## Discussion

It is important to obtain a wide range of candidate proteins in the biomarker discovery stage, because most candidates fail to be verified in a large number of clinical samples. Global data mining can reduce the time and cost in identifying candidates for clinical verification. The 5 data mining categories enabled us to screen frequently reported candidate genes and proteins.

In our study, 50 of 4658 candidate proteins, obtained from the 5-category data mining, were selected, based on frequency. Consequently, 28 of 50 candidates were detected in pooled serum, 19 of which were differentially expressed between the 3 groups. After individual serum MRM analysis using 36 healthy control, 18 before HCC treatment, and 18 after HCC treatment samples, 9 candidates had identical expression patterns by MRM analysis using serum that was pooled from the 3 groups, and 4 proteins were verified by western blot. By LR analysis, a 2-marker panel (ANLN and FLNB) was constructed, showing enhanced discriminatory power compared with AFP, with an AUC of 0.981 (healthy control versus before HCC treatment). Notably, 2 genes–ANLN and FLNB–were detected in the microarray data [25], [27] Thus, global data mining-based MRM verification, combined with multivariate analysis, is a robust method of developing HCC multimarkers.

AFP is the most useful tumor marker for HCC and is produced by immature cells of the fetus. Newborns have AFP levels of up to 3 g/L until age 18 months, when AFP levels begin to drop below 10 µg/L, which are maintained in adulthood. AFP levels in normal adults are approximately 5–10 µg/L, which liver cancer patients usually exceed. Levels of AFP exceed 50 µg/L in 40% to 60% of HCC patients [39], and the false negative rate for HCC diagnosis solely with AFP is 20% to 30%. When AFP levels exceed 500 µg/L, it would be detectable for changes in body.

Conversely, because AFP levels in blood are high in only approximately 60% of liver cancer patients and other benign diseases (hepatitis, liver cirrhosis), there is a limitation in using AFP alone as an HCC marker in blood [40], [41], [42], [43], [44], [45], [46], [47], [48]. In particular, in our western blot analysis, AFP levels differed significantly between the healthy control and before HCC treatment groups but not between the before HCC treatment and after HCC treatment groups.

The reproducibility with regard to experimental and analytical variation is a major goal of MRM analysis. To minimize the variation in MRM analysis, we generated 3 pooled samples, corresponding to the 3 groups (36 healthy control, 18 untreated HCC, and 18 treated HCC samples), and the 333 transitions that corresponded to 111 detectable peptides were monitored using MRM 5 times, in which the CV of all transition peak areas in the 3 groups was calculated. The transitions that had a CV% below 30% in all 3 groups were chosen as the final target transitions. Next, for individual MRM analysis, we selected only transitions that had a CV below 30%, as described in Supplementary Figure S1.

We have demonstrated the value of our scheme in selecting candidate proteins by 5-cartegory global data mining and verification of the candidate proteins by clinical MRM to develop HCC markers in blood. Further, our multimarker panel has improved discriminatory power compared with single protein markers, such as AFP. Our 2-marker panel, comprising ANLN and FNAB, distinguishes healthy controls from before HCC treatment patients better than AFP. Thus, we propose that this strategy–combining global data mining to screen candidates and verification by clinical MRM–is a robust, effective pipeline for HCC marker development than can be applied to markers of other diseases.

## Supporting Information

Figure S1 **Scatter plots of MRM quantitation data using pooling serum and individual serum from healthy control group, before HCC treatment group, and after HCC treatment group.** Left panel represents pooled serum from healthy control group, before HCC treatment group, and after HCC treatment group. Error bars represent the standard deviations from 5 technical replicates. Horizontal bars indicate the average serum level of the protein; *P*-values were calculated by ANOVA. Right panels indicate individual samples. See also Supplementary Table S4 and Supplementary Figure S2.
(PPTX)

Figure S2 **Interactive plots and AUC values for nine verified candidate biomarkers.** The normalized peak areas of transitions were compared between the healthy control group and before HCC treatment group and between the before HCC treatment group and after HCC treatment group. The interactive plots and ROC curves are represented by the transition peak areas of the 9 proteins. Interactive plots of each target peptide were extrapolated versus the standard peptide with regard to relative concentration, sensitivity, and specificity. See also Table 2.
(PPTX)

Table S1 **Clinical specimen information on liver cancer patients after the treatment.**
(XLSX)

Table S2 **Lists of candidate proteins obtained from global data mining.** Global data mining covers proteomics, cDNA microarray, copy number variation, and somatic mutation. Each number such as 0 and 1 in the cell represents hit count of candidate protein.
(XLSX)

Table S3 **The list of potential biomarkers was filtered step by step per the verification steps.**
(XLSX)

Table S4 **Results of technical reproducibility was examined using pooled serum.** Also see Figure S1.
(XLSX)

Table S5 **List of peptides and fragment ions for the analyzed proteins.**
(XLSX)

Table S6 **Tolerances and Variance inflation factors (VIFs) of collinearity analysis for 2 models.**
(XLSX)

Table S7 **Classification tables from logistic regression models (Cross validated, Leave-one out).**
(XLSX)
