# Supplementary material for: Development of Biomarkers for Screening Hepatocellular Carcinoma Using Global Data Mining and Multiple Reaction Monitoring
Source: PLoS One. 2013 May 22;8(5):e63468. doi: 10.1371/journal.pone.0063468 (PMC3661589; doi:10.1371/journal.pone.0063468)
Supplement: Figure S1 — Scatter plots of MRM quantitation data using pooling serum and individual serum from healthy control group, before HCC treatment group, and after HCC treatment group. Left panel represents pooled serum from healthy control group, before HCC treatment group, and after HCC treatment group. Error bars represent the standard deviations from 5 technical replicates. Horizontal bars indicate the average serum level of the protein; P-values were calculated by ANOVA. Right panels indicate individual samples. See also Supplementary Table S4 and Supplementary Figure S2. (PPTX) [file pone.0063468.s001.pptx]

## Slide 1
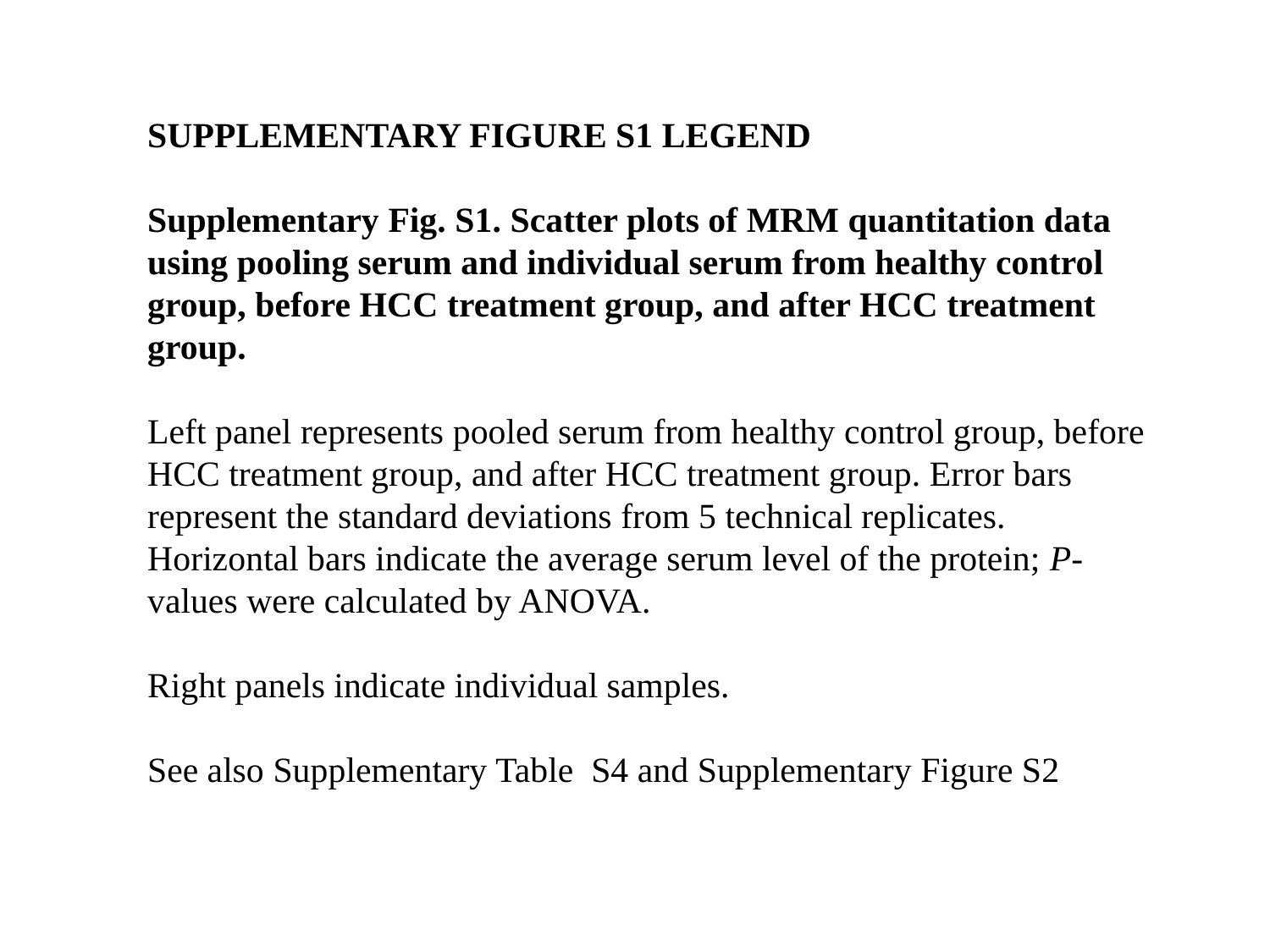

Supplementary Figure S1 legend
Supplementary Fig. S1. Scatter plots of MRM quantitation data using pooling serum and individual serum from healthy control group, before HCC treatment group, and after HCC treatment group.
Left panel represents pooled serum from healthy control group, before HCC treatment group, and after HCC treatment group. Error bars represent the standard deviations from 5 technical replicates. Horizontal bars indicate the average serum level of the protein; P-values were calculated by ANOVA.
Right panels indicate individual samples.
See also Supplementary Table S4 and Supplementary Figure S2

## Slide 2
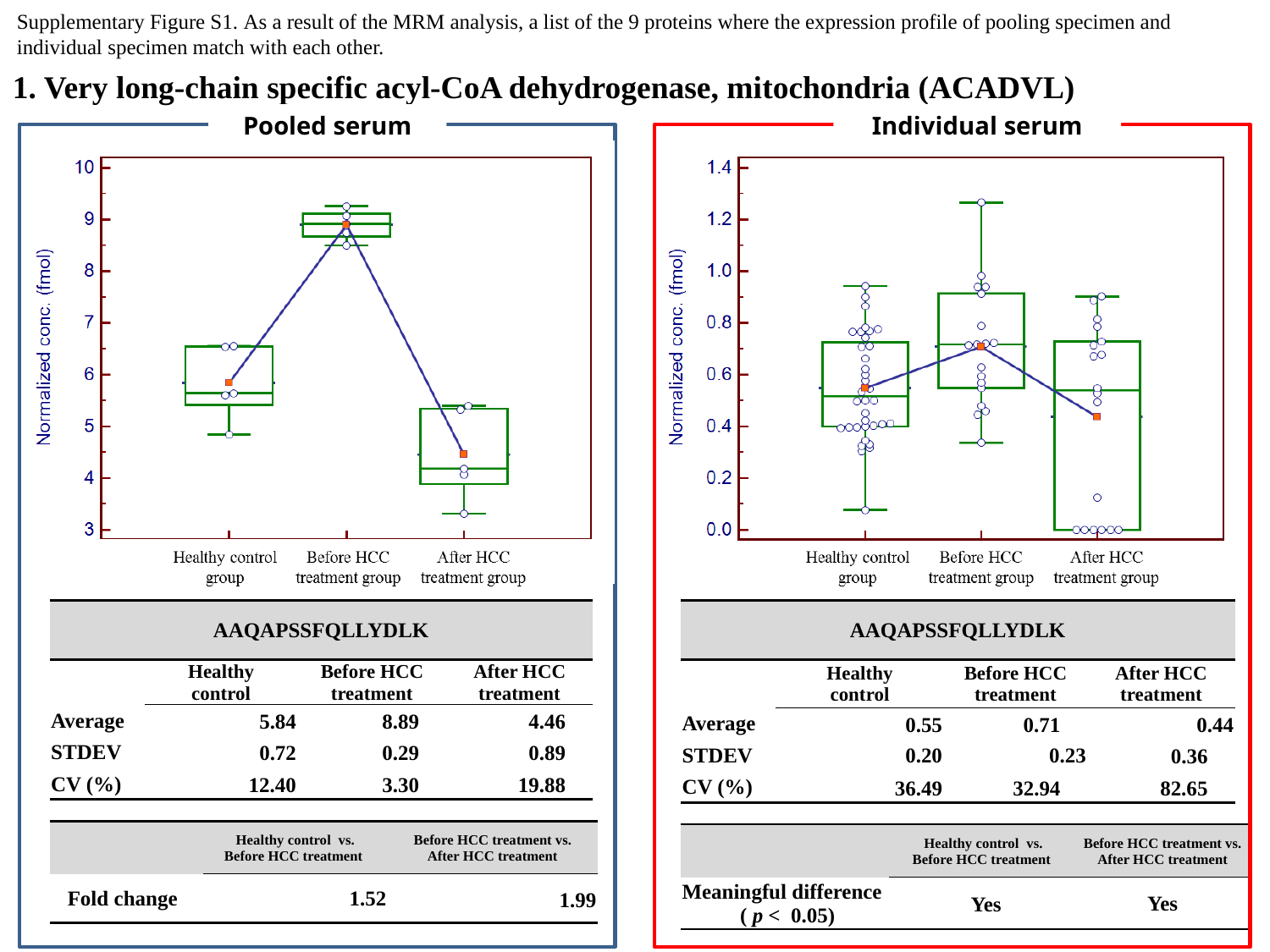

Supplementary Figure S1. As a result of the MRM analysis, a list of the 9 proteins where the expression profile of pooling specimen and individual specimen match with each other.
1. Very long-chain specific acyl-CoA dehydrogenase, mitochondria (ACADVL)
Pooled serum
Individual serum
| AAQAPSSFQLLYDLK | | | |
| --- | --- | --- | --- |
| | Healthy control | Before HCC treatment | After HCC treatment |
| Average | 5.84 | 8.89 | 4.46 |
| STDEV | 0.72 | 0.29 | 0.89 |
| CV (%) | 12.40 | 3.30 | 19.88 |
| AAQAPSSFQLLYDLK | | | |
| --- | --- | --- | --- |
| | Healthy control | Before HCC treatment | After HCC treatment |
| Average | 0.55 | 0.71 | 0.44 |
| STDEV | 0.20 | 0.23 | 0.36 |
| CV (%) | 36.49 | 32.94 | 82.65 |
| | | Healthy control vs. Before HCC treatment | Before HCC treatment vs. After HCC treatment |
| --- | --- | --- | --- |
| Fold change | 1.52 | | 1.99 |
| | Healthy control vs. Before HCC treatment | Before HCC treatment vs. After HCC treatment |
| --- | --- | --- |
| Meaningful difference ( p < 0.05) | Yes | Yes |

## Slide 3
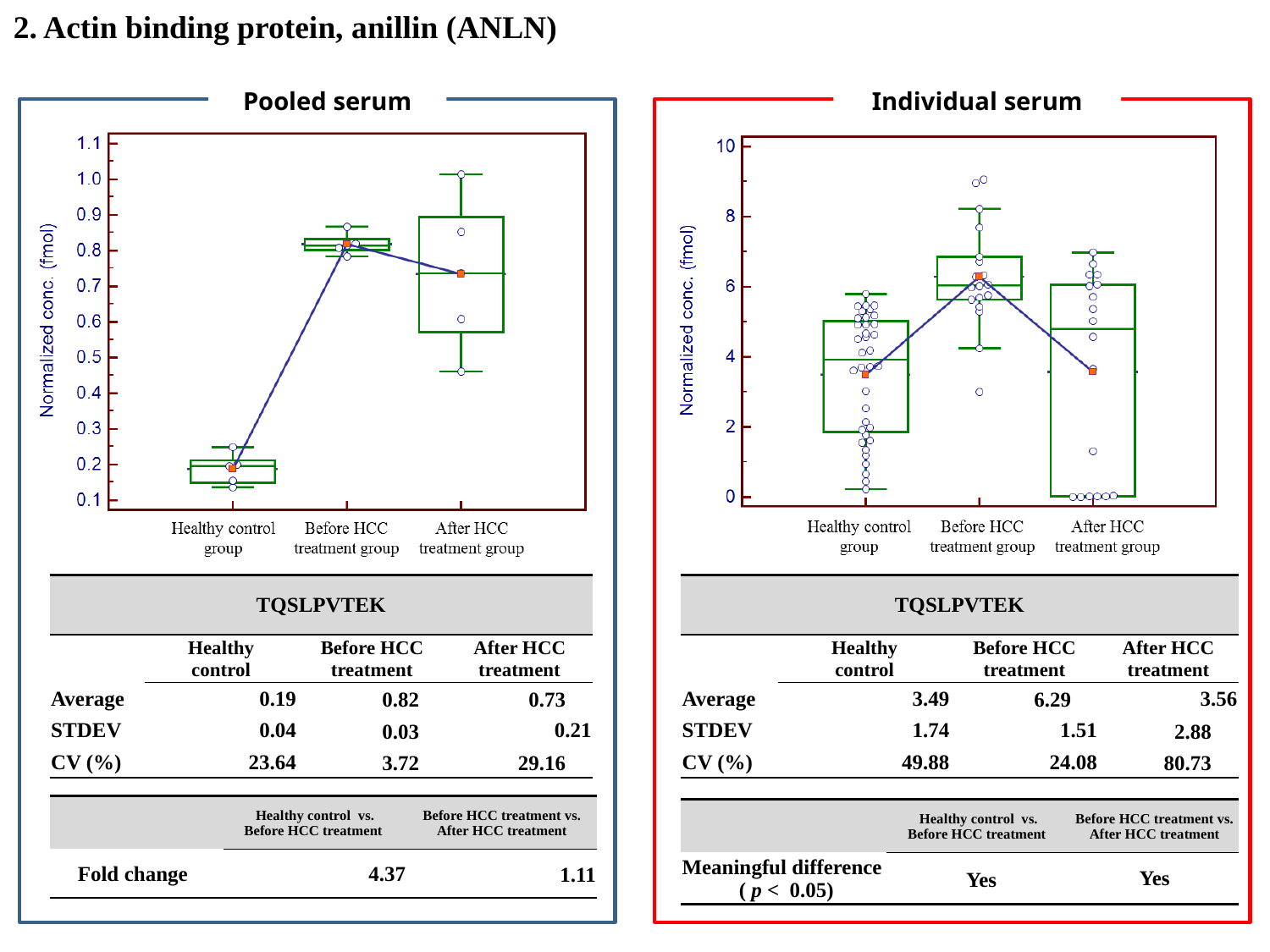

2. Actin binding protein, anillin (ANLN)
Pooled serum
Individual serum
| TQSLPVTEK | | | |
| --- | --- | --- | --- |
| | Healthy control | Before HCC treatment | After HCC treatment |
| Average | 0.19 | 0.82 | 0.73 |
| STDEV | 0.04 | 0.03 | 0.21 |
| CV (%) | 23.64 | 3.72 | 29.16 |
| TQSLPVTEK | | | |
| --- | --- | --- | --- |
| | Healthy control | Before HCC treatment | After HCC treatment |
| Average | 3.49 | 6.29 | 3.56 |
| STDEV | 1.74 | 1.51 | 2.88 |
| CV (%) | 49.88 | 24.08 | 80.73 |
| | | Healthy control vs. Before HCC treatment | Before HCC treatment vs. After HCC treatment |
| --- | --- | --- | --- |
| Fold change | 4.37 | | 1.11 |
| | Healthy control vs. Before HCC treatment | Before HCC treatment vs. After HCC treatment |
| --- | --- | --- |
| Meaningful difference ( p < 0.05) | Yes | Yes |

## Slide 4
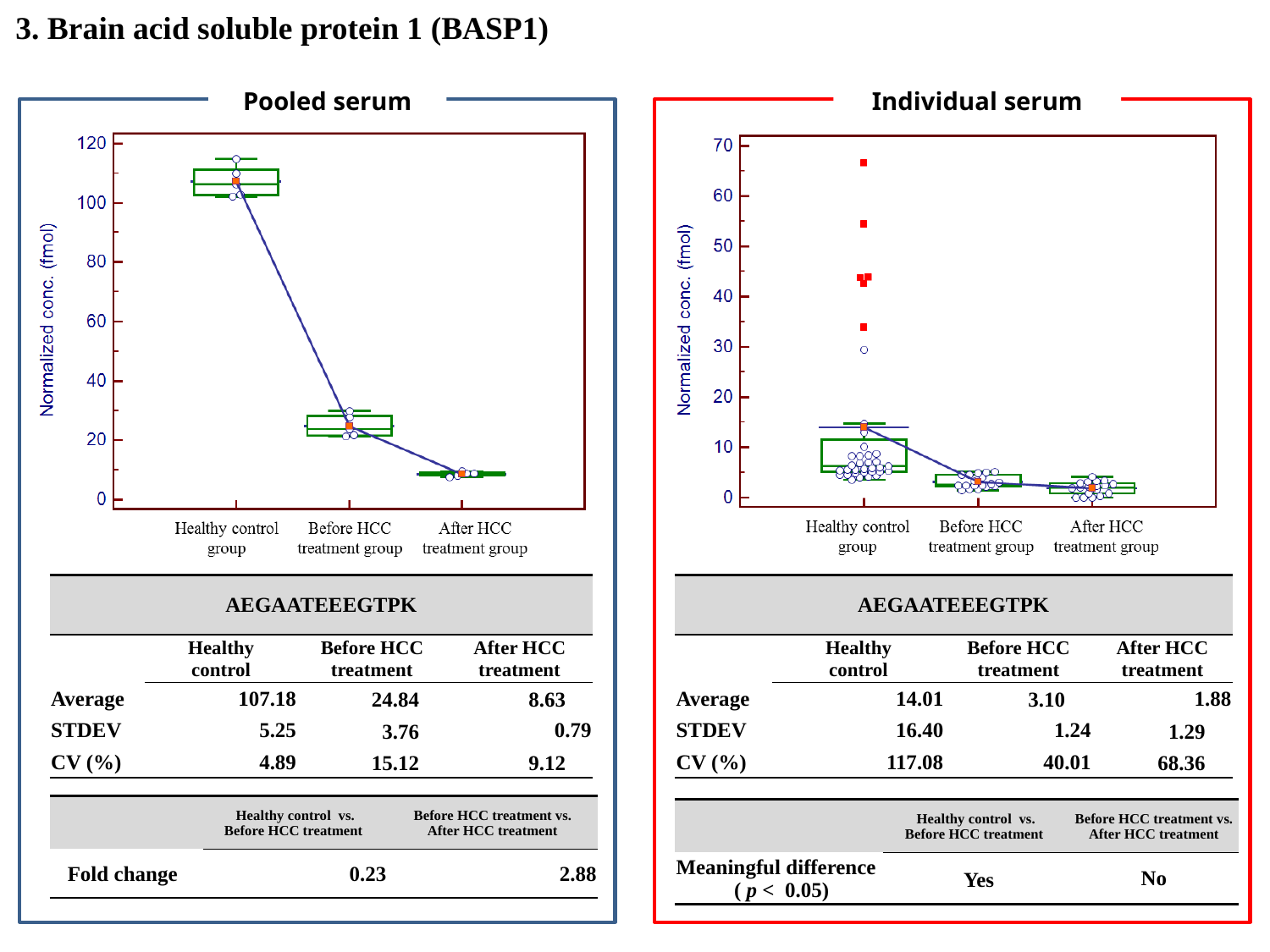

3. Brain acid soluble protein 1 (BASP1)
Pooled serum
Individual serum
| AEGAATEEEGTPK | | | |
| --- | --- | --- | --- |
| | Healthy control | Before HCC treatment | After HCC treatment |
| Average | 107.18 | 24.84 | 8.63 |
| STDEV | 5.25 | 3.76 | 0.79 |
| CV (%) | 4.89 | 15.12 | 9.12 |
| AEGAATEEEGTPK | | | |
| --- | --- | --- | --- |
| | Healthy control | Before HCC treatment | After HCC treatment |
| Average | 14.01 | 3.10 | 1.88 |
| STDEV | 16.40 | 1.24 | 1.29 |
| CV (%) | 117.08 | 40.01 | 68.36 |
| | | Healthy control vs. Before HCC treatment | Before HCC treatment vs. After HCC treatment |
| --- | --- | --- | --- |
| Fold change | 0.23 | | 2.88 |
| | Healthy control vs. Before HCC treatment | Before HCC treatment vs. After HCC treatment |
| --- | --- | --- |
| Meaningful difference ( p < 0.05) | Yes | No |

## Slide 5
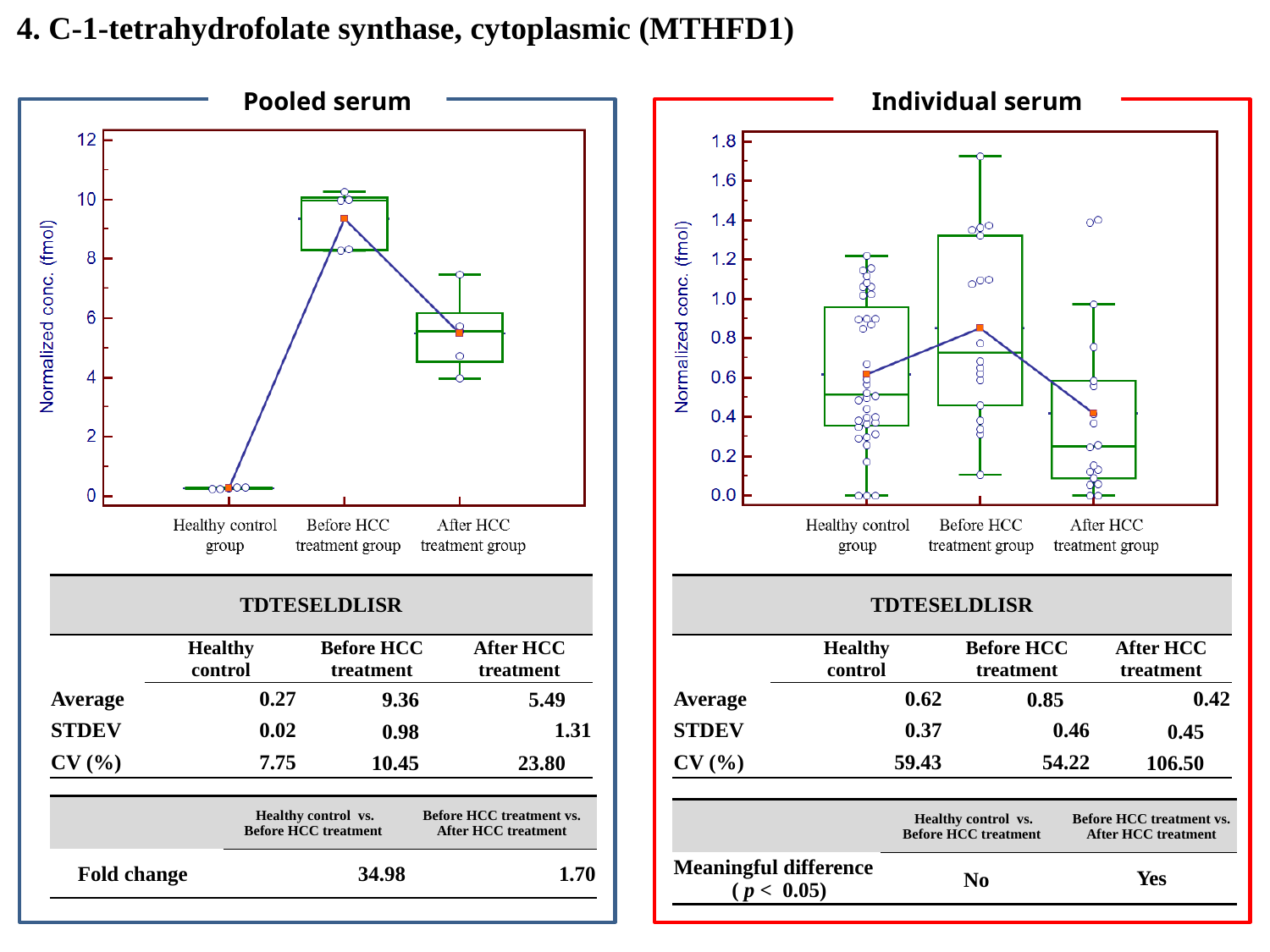

4. C-1-tetrahydrofolate synthase, cytoplasmic (MTHFD1)
Pooled serum
Individual serum
| TDTESELDLISR | | | |
| --- | --- | --- | --- |
| | Healthy control | Before HCC treatment | After HCC treatment |
| Average | 0.27 | 9.36 | 5.49 |
| STDEV | 0.02 | 0.98 | 1.31 |
| CV (%) | 7.75 | 10.45 | 23.80 |
| TDTESELDLISR | | | |
| --- | --- | --- | --- |
| | Healthy control | Before HCC treatment | After HCC treatment |
| Average | 0.62 | 0.85 | 0.42 |
| STDEV | 0.37 | 0.46 | 0.45 |
| CV (%) | 59.43 | 54.22 | 106.50 |
| | | Healthy control vs. Before HCC treatment | Before HCC treatment vs. After HCC treatment |
| --- | --- | --- | --- |
| Fold change | 34.98 | | 1.70 |
| | Healthy control vs. Before HCC treatment | Before HCC treatment vs. After HCC treatment |
| --- | --- | --- |
| Meaningful difference ( p < 0.05) | No | Yes |

## Slide 6
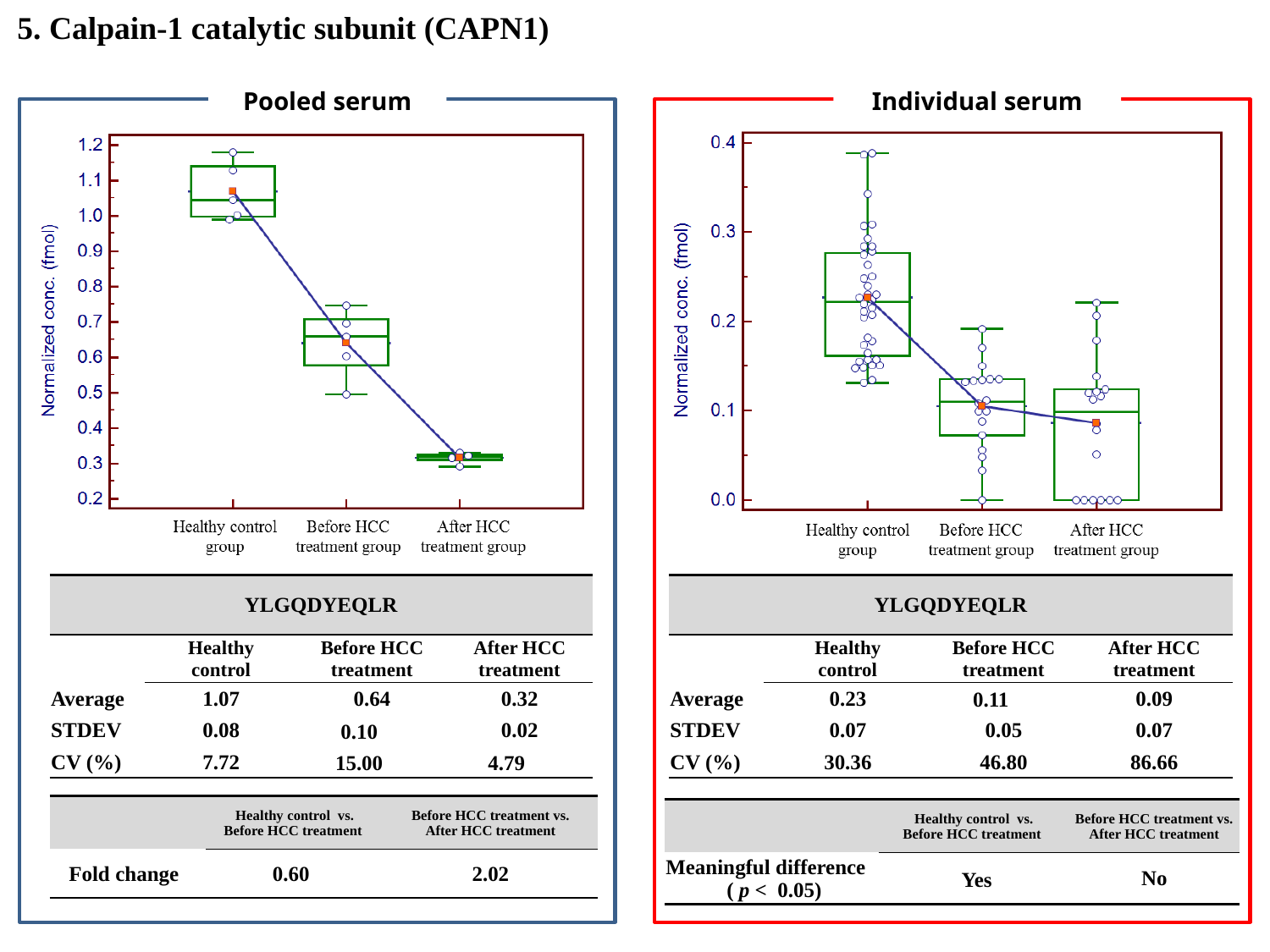

5. Calpain-1 catalytic subunit (CAPN1)
Pooled serum
Individual serum
| YLGQDYEQLR | | | |
| --- | --- | --- | --- |
| | Healthy control | Before HCC treatment | After HCC treatment |
| Average | 1.07 | 0.64 | 0.32 |
| STDEV | 0.08 | 0.10 | 0.02 |
| CV (%) | 7.72 | 15.00 | 4.79 |
| YLGQDYEQLR | | | |
| --- | --- | --- | --- |
| | Healthy control | Before HCC treatment | After HCC treatment |
| Average | 0.23 | 0.11 | 0.09 |
| STDEV | 0.07 | 0.05 | 0.07 |
| CV (%) | 30.36 | 46.80 | 86.66 |
| | | Healthy control vs. Before HCC treatment | Before HCC treatment vs. After HCC treatment |
| --- | --- | --- | --- |
| Fold change | 0.60 | | 2.02 |
| | Healthy control vs. Before HCC treatment | Before HCC treatment vs. After HCC treatment |
| --- | --- | --- |
| Meaningful difference ( p < 0.05) | Yes | No |

## Slide 7
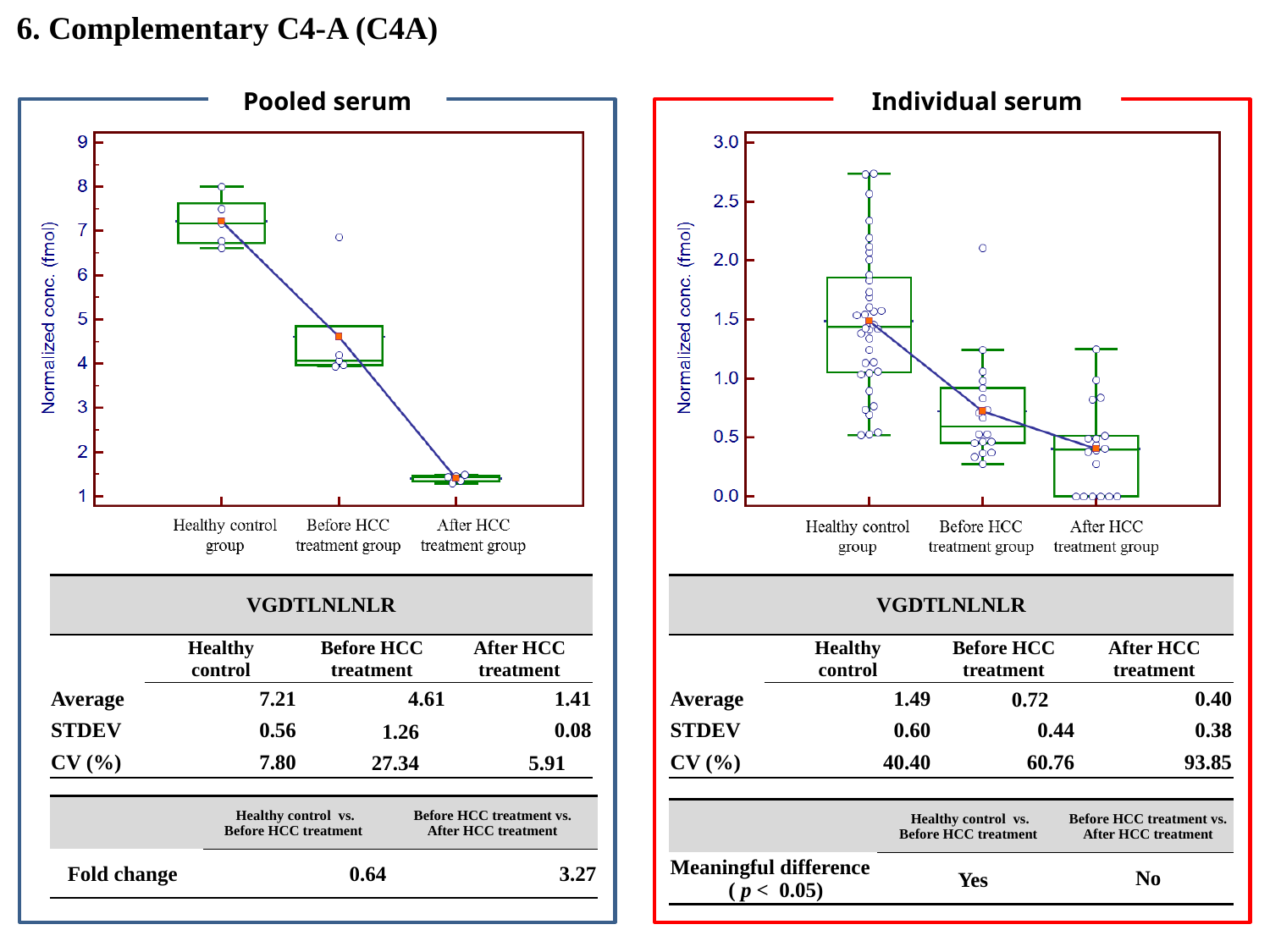

6. Complementary C4-A (C4A)
Pooled serum
Individual serum
| VGDTLNLNLR | | | |
| --- | --- | --- | --- |
| | Healthy control | Before HCC treatment | After HCC treatment |
| Average | 7.21 | 4.61 | 1.41 |
| STDEV | 0.56 | 1.26 | 0.08 |
| CV (%) | 7.80 | 27.34 | 5.91 |
| VGDTLNLNLR | | | |
| --- | --- | --- | --- |
| | Healthy control | Before HCC treatment | After HCC treatment |
| Average | 1.49 | 0.72 | 0.40 |
| STDEV | 0.60 | 0.44 | 0.38 |
| CV (%) | 40.40 | 60.76 | 93.85 |
| | | Healthy control vs. Before HCC treatment | Before HCC treatment vs. After HCC treatment |
| --- | --- | --- | --- |
| Fold change | 0.64 | | 3.27 |
| | Healthy control vs. Before HCC treatment | Before HCC treatment vs. After HCC treatment |
| --- | --- | --- |
| Meaningful difference ( p < 0.05) | Yes | No |

## Slide 8
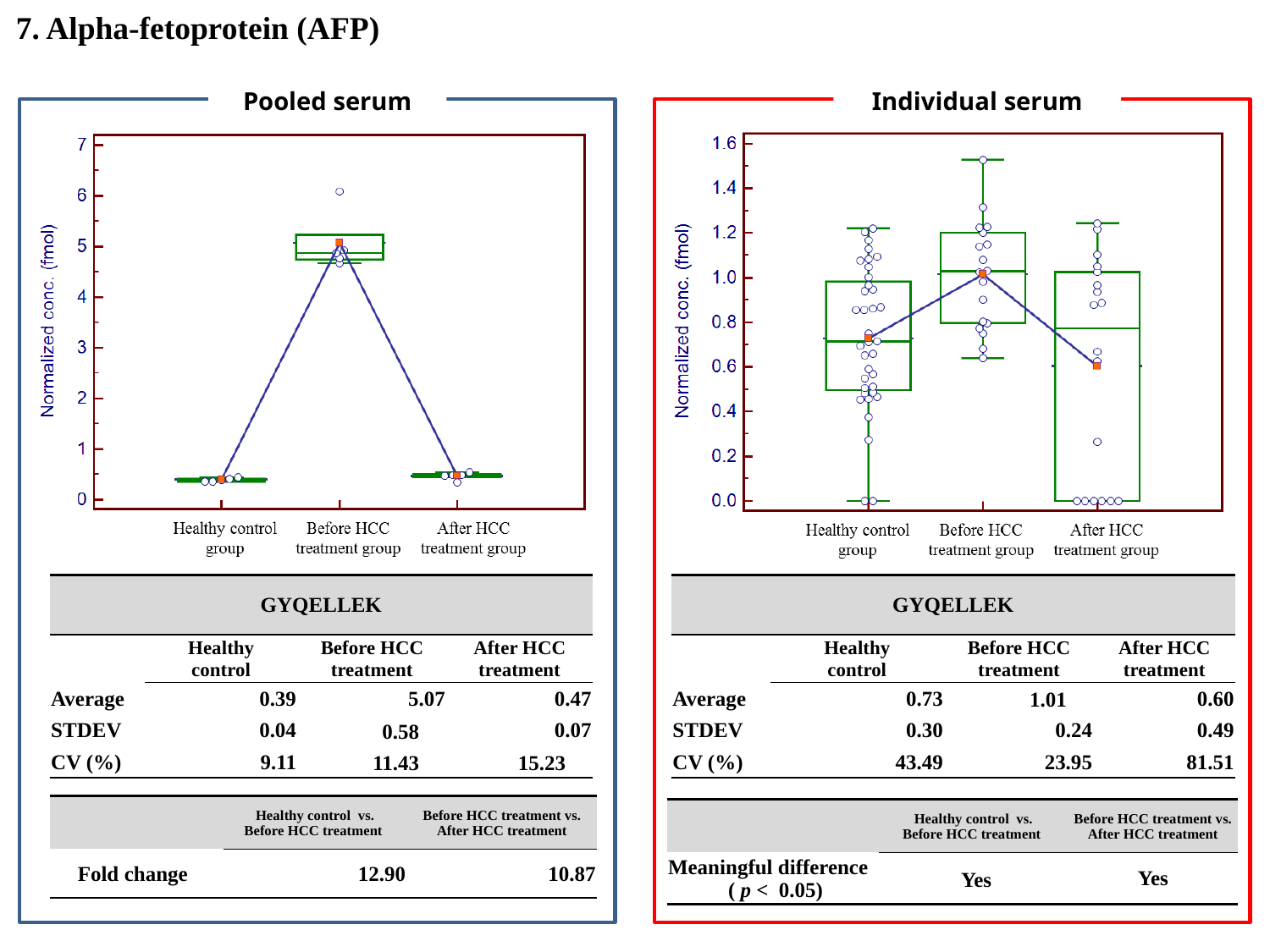

7. Alpha-fetoprotein (AFP)
Pooled serum
Individual serum
| GYQELLEK | | | |
| --- | --- | --- | --- |
| | Healthy control | Before HCC treatment | After HCC treatment |
| Average | 0.39 | 5.07 | 0.47 |
| STDEV | 0.04 | 0.58 | 0.07 |
| CV (%) | 9.11 | 11.43 | 15.23 |
| GYQELLEK | | | |
| --- | --- | --- | --- |
| | Healthy control | Before HCC treatment | After HCC treatment |
| Average | 0.73 | 1.01 | 0.60 |
| STDEV | 0.30 | 0.24 | 0.49 |
| CV (%) | 43.49 | 23.95 | 81.51 |
| | | Healthy control vs. Before HCC treatment | Before HCC treatment vs. After HCC treatment |
| --- | --- | --- | --- |
| Fold change | 12.90 | | 10.87 |
| | Healthy control vs. Before HCC treatment | Before HCC treatment vs. After HCC treatment |
| --- | --- | --- |
| Meaningful difference ( p < 0.05) | Yes | Yes |

## Slide 9
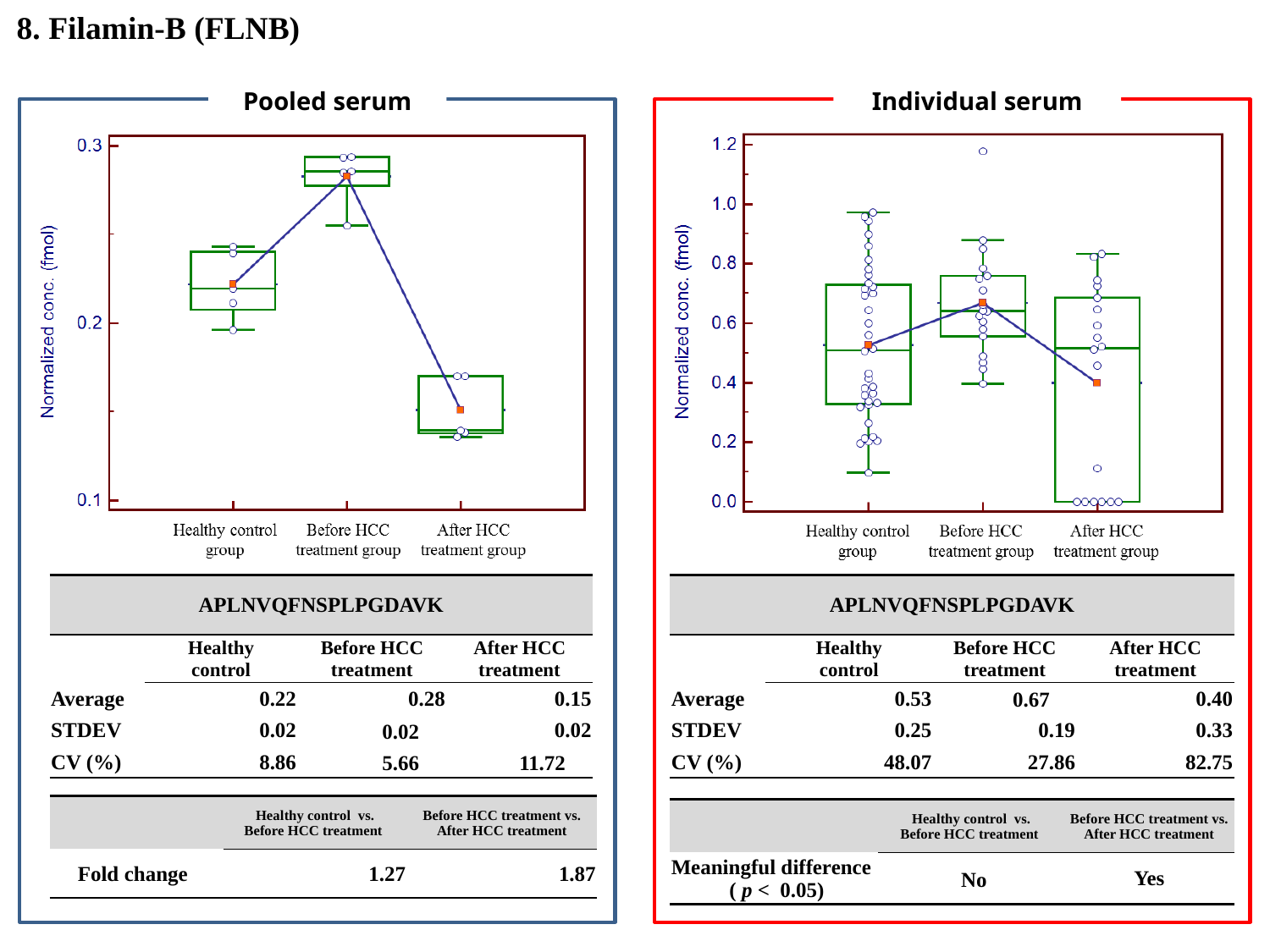

8. Filamin-B (FLNB)
Pooled serum
Individual serum
| APLNVQFNSPLPGDAVK | | | |
| --- | --- | --- | --- |
| | Healthy control | Before HCC treatment | After HCC treatment |
| Average | 0.22 | 0.28 | 0.15 |
| STDEV | 0.02 | 0.02 | 0.02 |
| CV (%) | 8.86 | 5.66 | 11.72 |
| APLNVQFNSPLPGDAVK | | | |
| --- | --- | --- | --- |
| | Healthy control | Before HCC treatment | After HCC treatment |
| Average | 0.53 | 0.67 | 0.40 |
| STDEV | 0.25 | 0.19 | 0.33 |
| CV (%) | 48.07 | 27.86 | 82.75 |
| | | Healthy control vs. Before HCC treatment | Before HCC treatment vs. After HCC treatment |
| --- | --- | --- | --- |
| Fold change | 1.27 | | 1.87 |
| | Healthy control vs. Before HCC treatment | Before HCC treatment vs. After HCC treatment |
| --- | --- | --- |
| Meaningful difference ( p < 0.05) | No | Yes |

## Slide 10
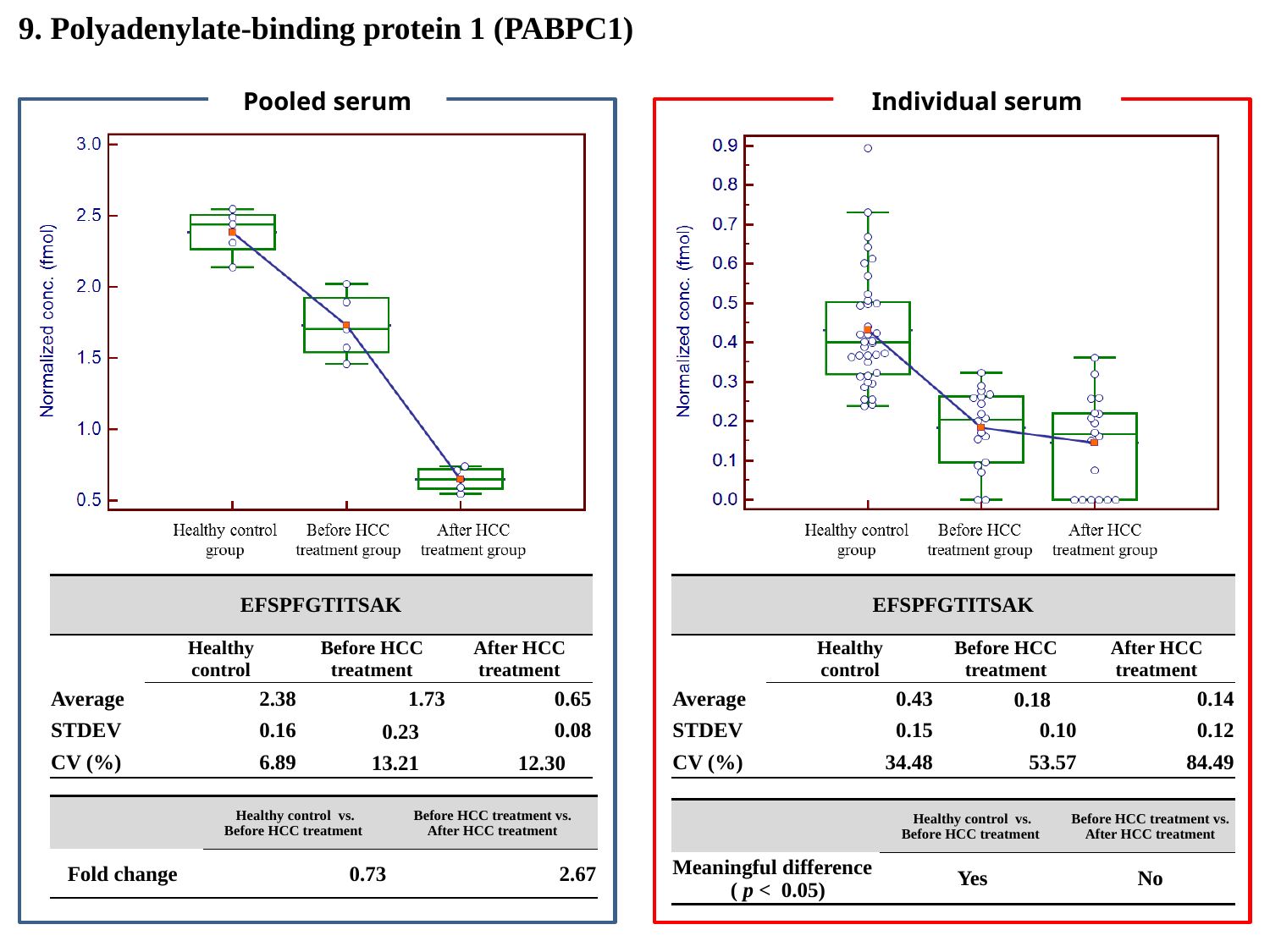

9. Polyadenylate-binding protein 1 (PABPC1)
Pooled serum
Individual serum
| EFSPFGTITSAK | | | |
| --- | --- | --- | --- |
| | Healthy control | Before HCC treatment | After HCC treatment |
| Average | 2.38 | 1.73 | 0.65 |
| STDEV | 0.16 | 0.23 | 0.08 |
| CV (%) | 6.89 | 13.21 | 12.30 |
| EFSPFGTITSAK | | | |
| --- | --- | --- | --- |
| | Healthy control | Before HCC treatment | After HCC treatment |
| Average | 0.43 | 0.18 | 0.14 |
| STDEV | 0.15 | 0.10 | 0.12 |
| CV (%) | 34.48 | 53.57 | 84.49 |
| | | Healthy control vs. Before HCC treatment | Before HCC treatment vs. After HCC treatment |
| --- | --- | --- | --- |
| Fold change | 0.73 | | 2.67 |
| | Healthy control vs. Before HCC treatment | Before HCC treatment vs. After HCC treatment |
| --- | --- | --- |
| Meaningful difference ( p < 0.05) | Yes | No |
