# Supplementary material for: Development of Biomarkers for Screening Hepatocellular Carcinoma Using Global Data Mining and Multiple Reaction Monitoring
Source: PLoS One. 2013 May 22;8(5):e63468. doi: 10.1371/journal.pone.0063468 (PMC3661589; doi:10.1371/journal.pone.0063468)
Supplement: Figure S2 — Interactive plots and AUC values for nine verified candidate biomarkers. The normalized peak areas of transitions were compared between the healthy control group and before HCC treatment group and between the before HCC treatment group and after HCC treatment group. The interactive plots and ROC curves are represented by the transition peak areas of the 9 proteins. Interactive plots of each target peptide were extrapolated versus the standard peptide with regard to relative concentration, sensitivity, and specificity. See also Table 2. (PPTX) [file pone.0063468.s002.pptx]

## Slide 1
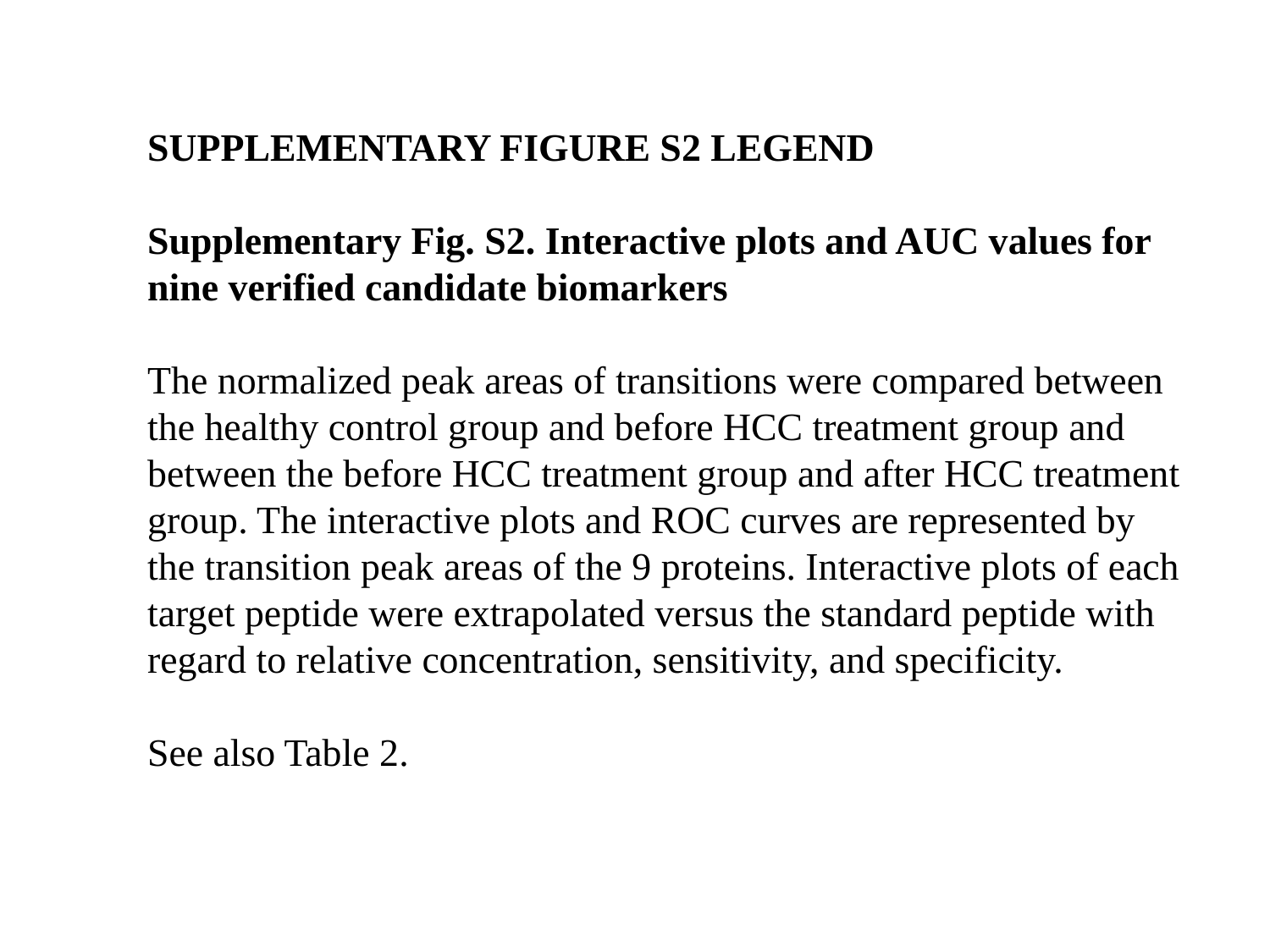

Supplementary Figure S2 legend
Supplementary Fig. S2. Interactive plots and AUC values for nine verified candidate biomarkers
The normalized peak areas of transitions were compared between the healthy control group and before HCC treatment group and between the before HCC treatment group and after HCC treatment group. The interactive plots and ROC curves are represented by the transition peak areas of the 9 proteins. Interactive plots of each target peptide were extrapolated versus the standard peptide with regard to relative concentration, sensitivity, and specificity.
See also Table 2.

## Slide 2
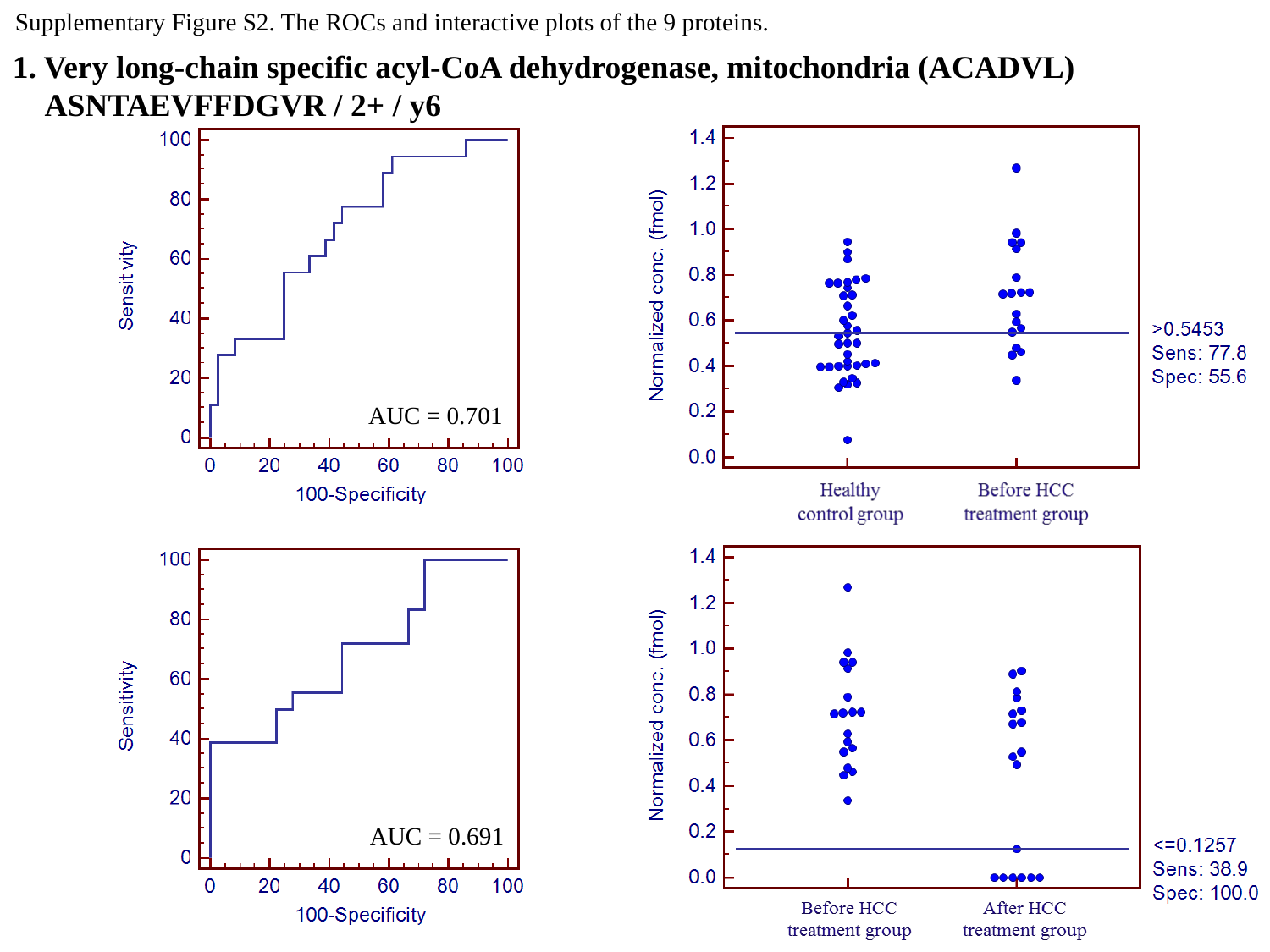

Supplementary Figure S2. The ROCs and interactive plots of the 9 proteins.
1. Very long-chain specific acyl-CoA dehydrogenase, mitochondria (ACADVL)
 ASNTAEVFFDGVR / 2+ / y6
AUC = 0.701
AUC = 0.691

## Slide 3
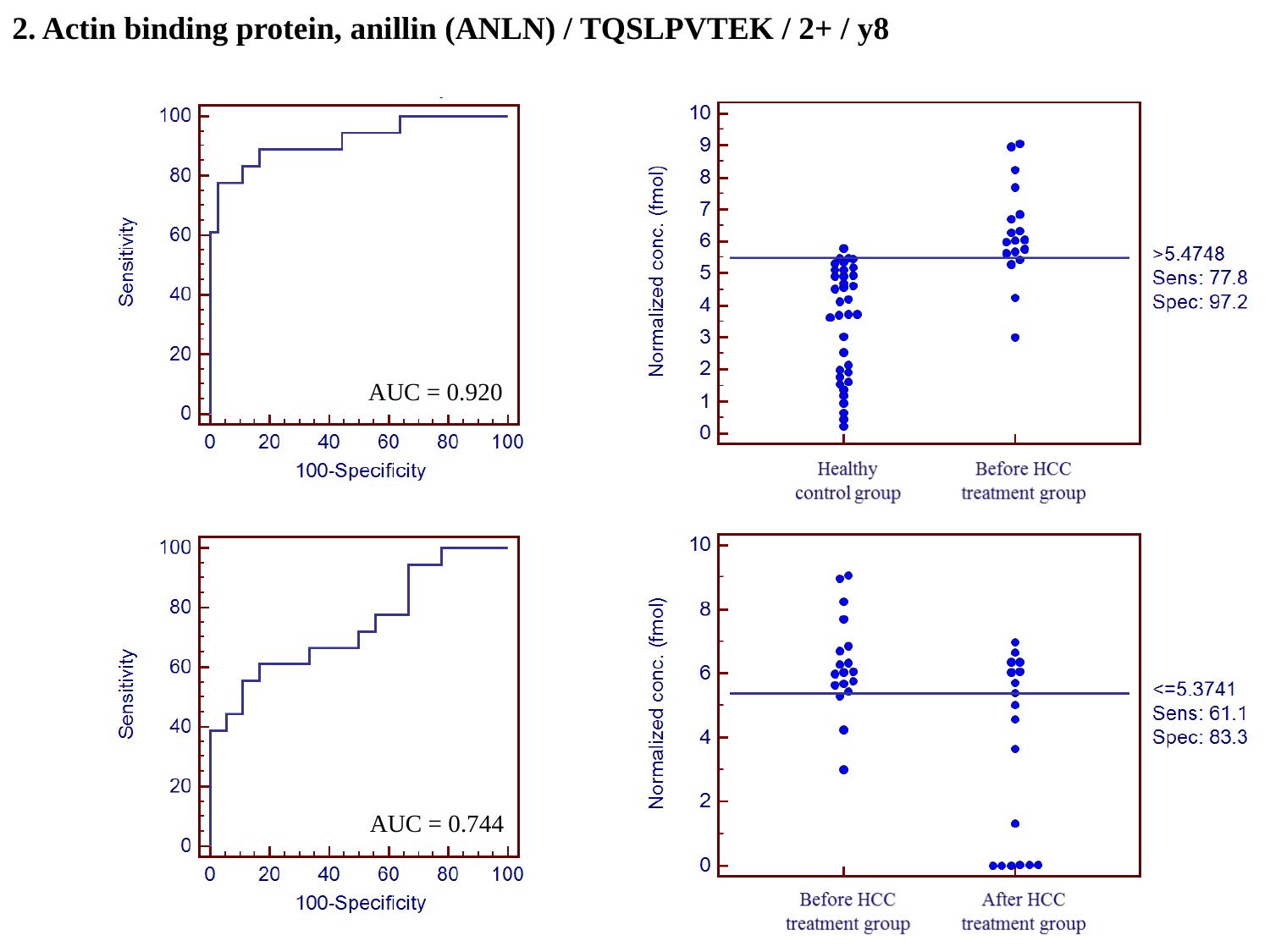

2. Actin binding protein, anillin (ANLN) / TQSLPVTEK / 2+ / y8
AUC = 0.920
AUC = 0.744

## Slide 4
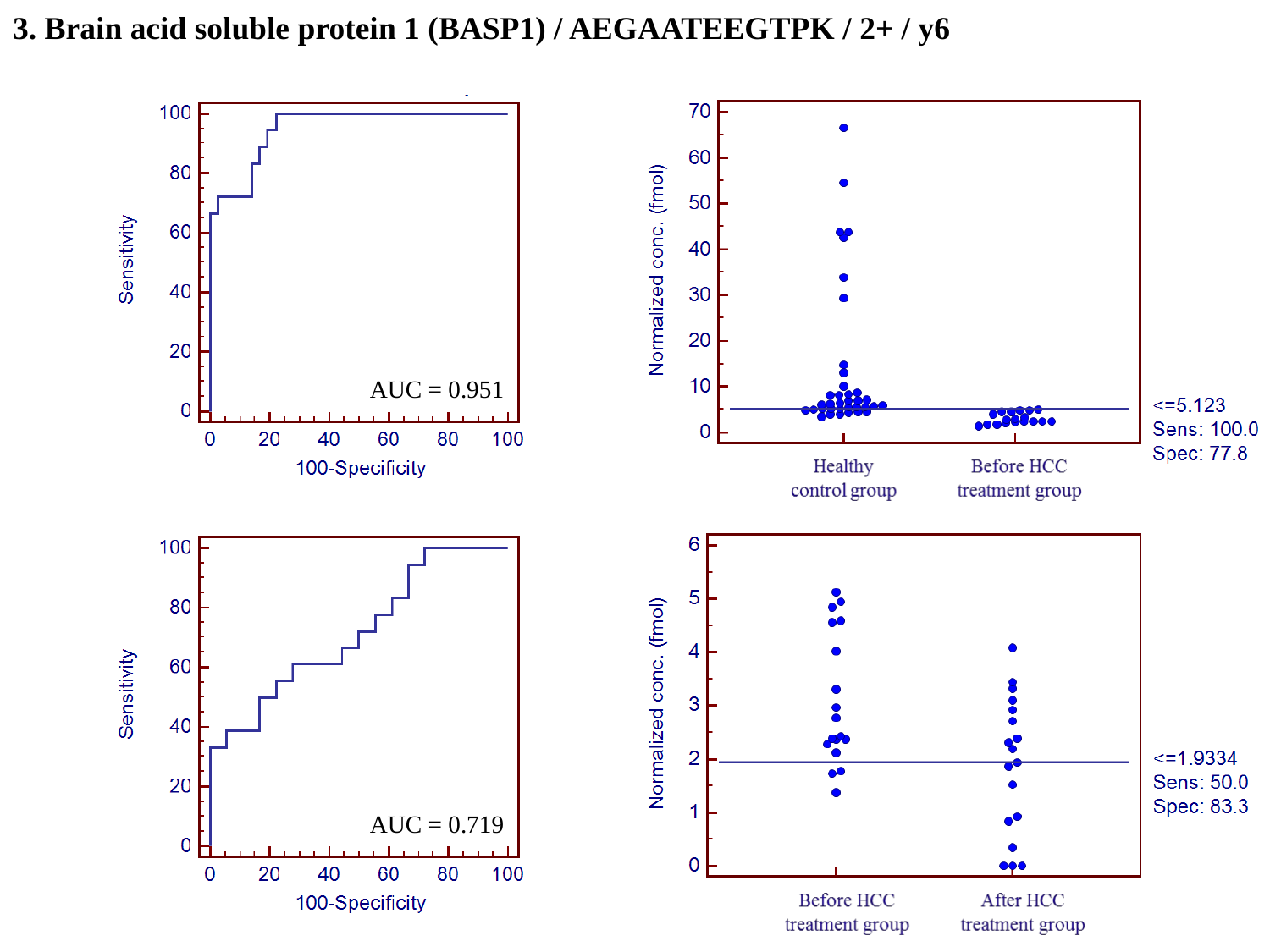

3. Brain acid soluble protein 1 (BASP1) / AEGAATEEGTPK / 2+ / y6
AUC = 0.951
AUC = 0.719

## Slide 5
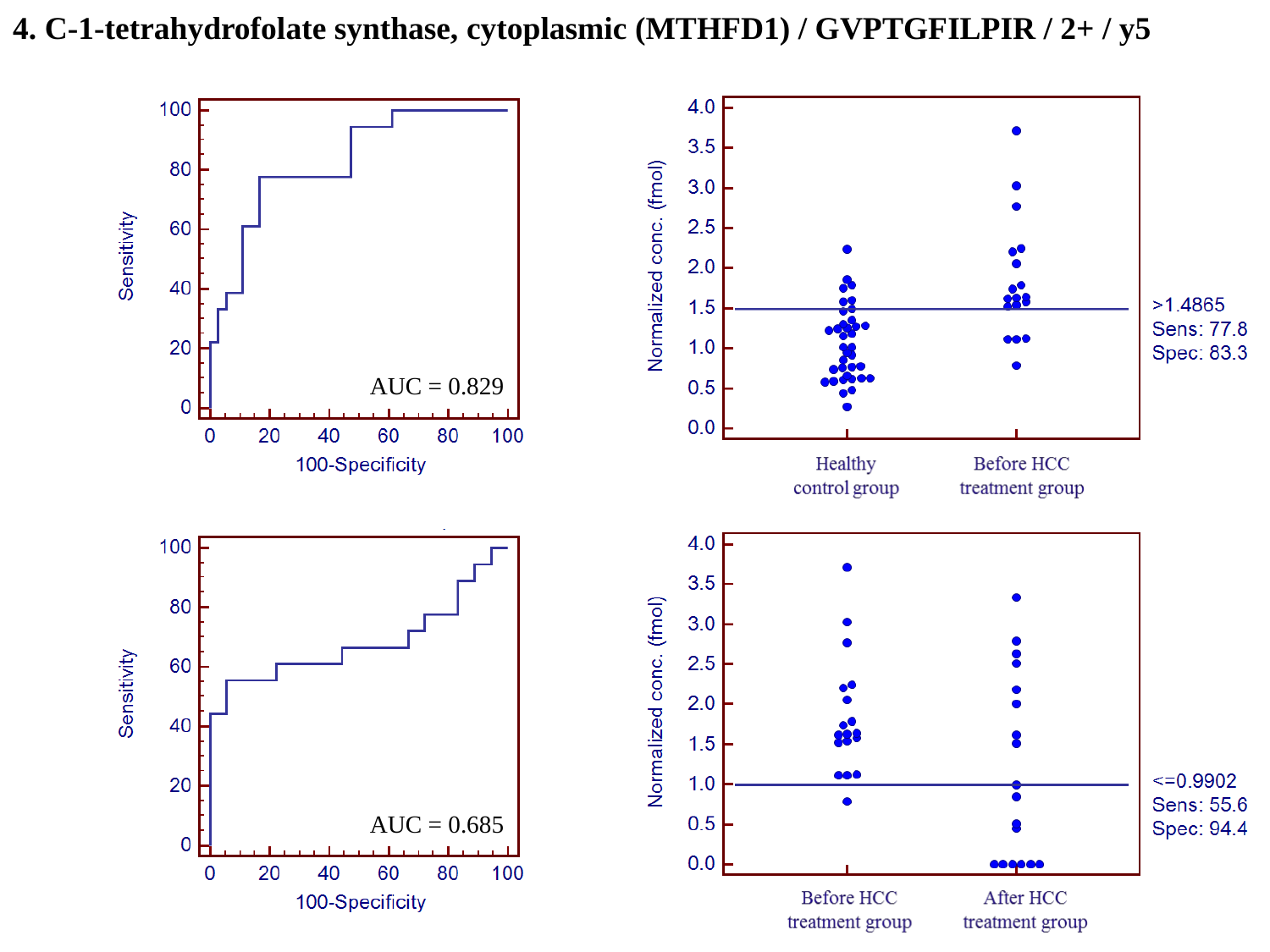

4. C-1-tetrahydrofolate synthase, cytoplasmic (MTHFD1) / GVPTGFILPIR / 2+ / y5
AUC = 0.829
AUC = 0.685

## Slide 6
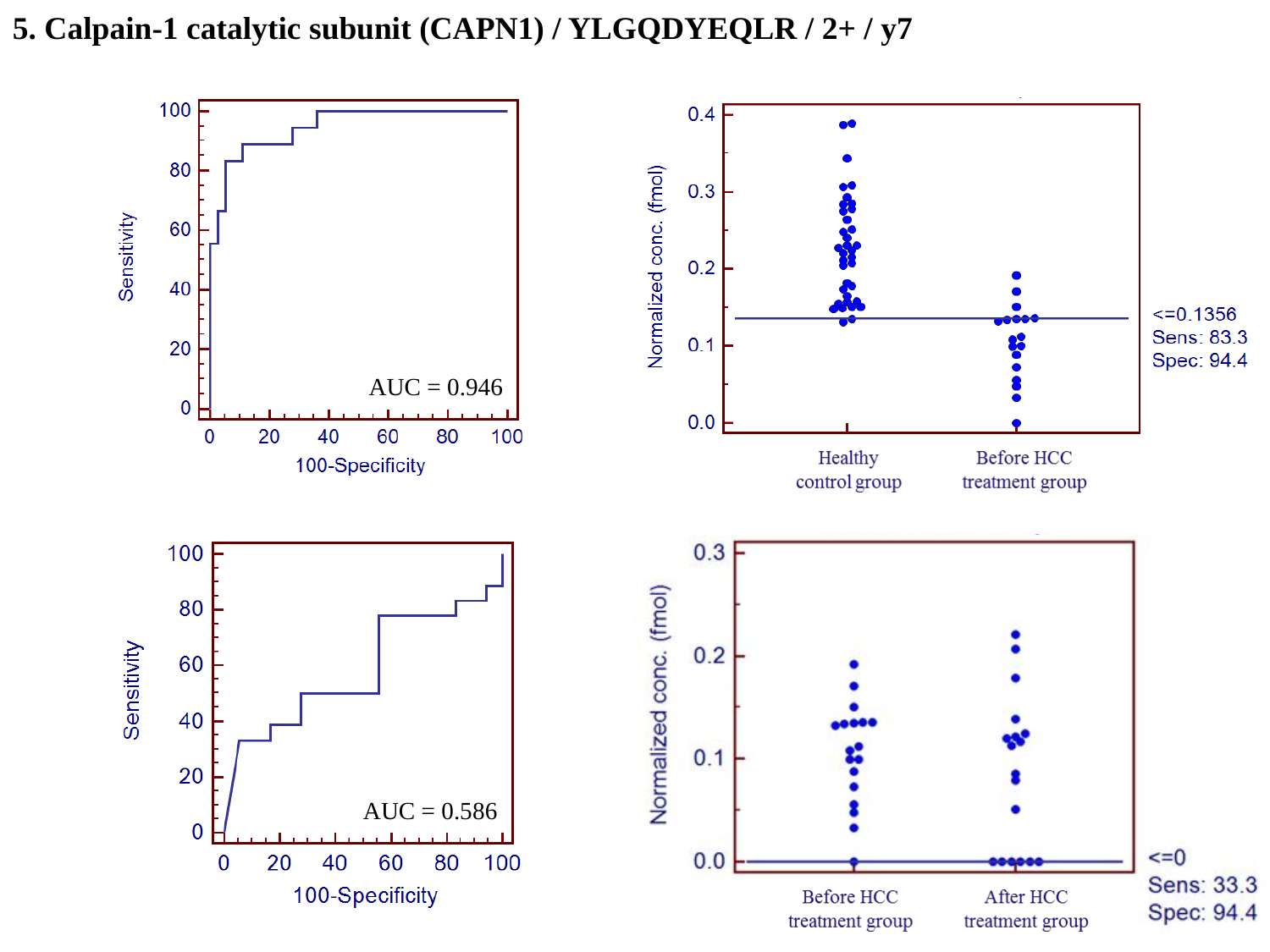

5. Calpain-1 catalytic subunit (CAPN1) / YLGQDYEQLR / 2+ / y7
AUC = 0.946
AUC = 0.586

## Slide 7
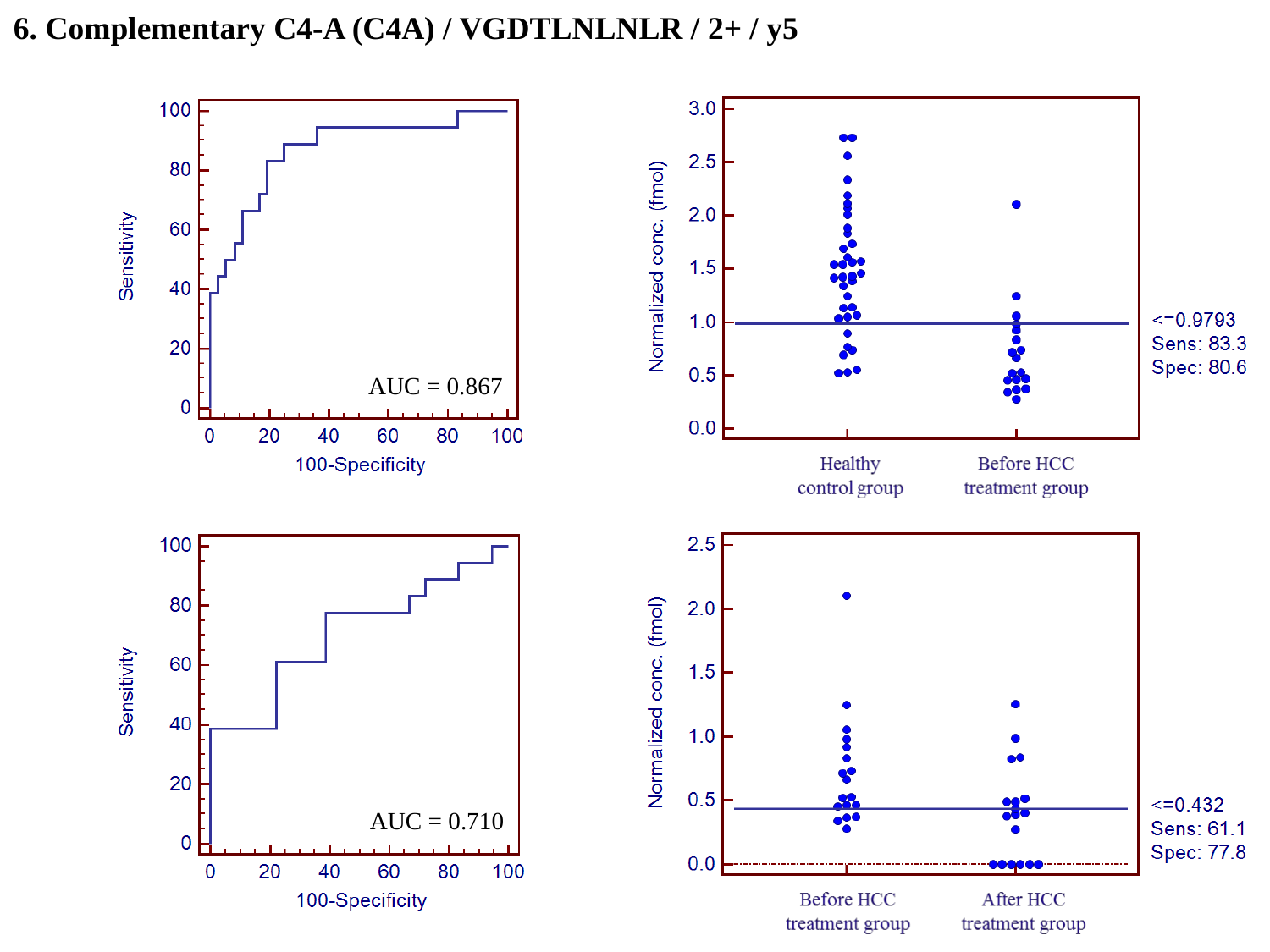

6. Complementary C4-A (C4A) / VGDTLNLNLR / 2+ / y5
AUC = 0.867
AUC = 0.710

## Slide 8
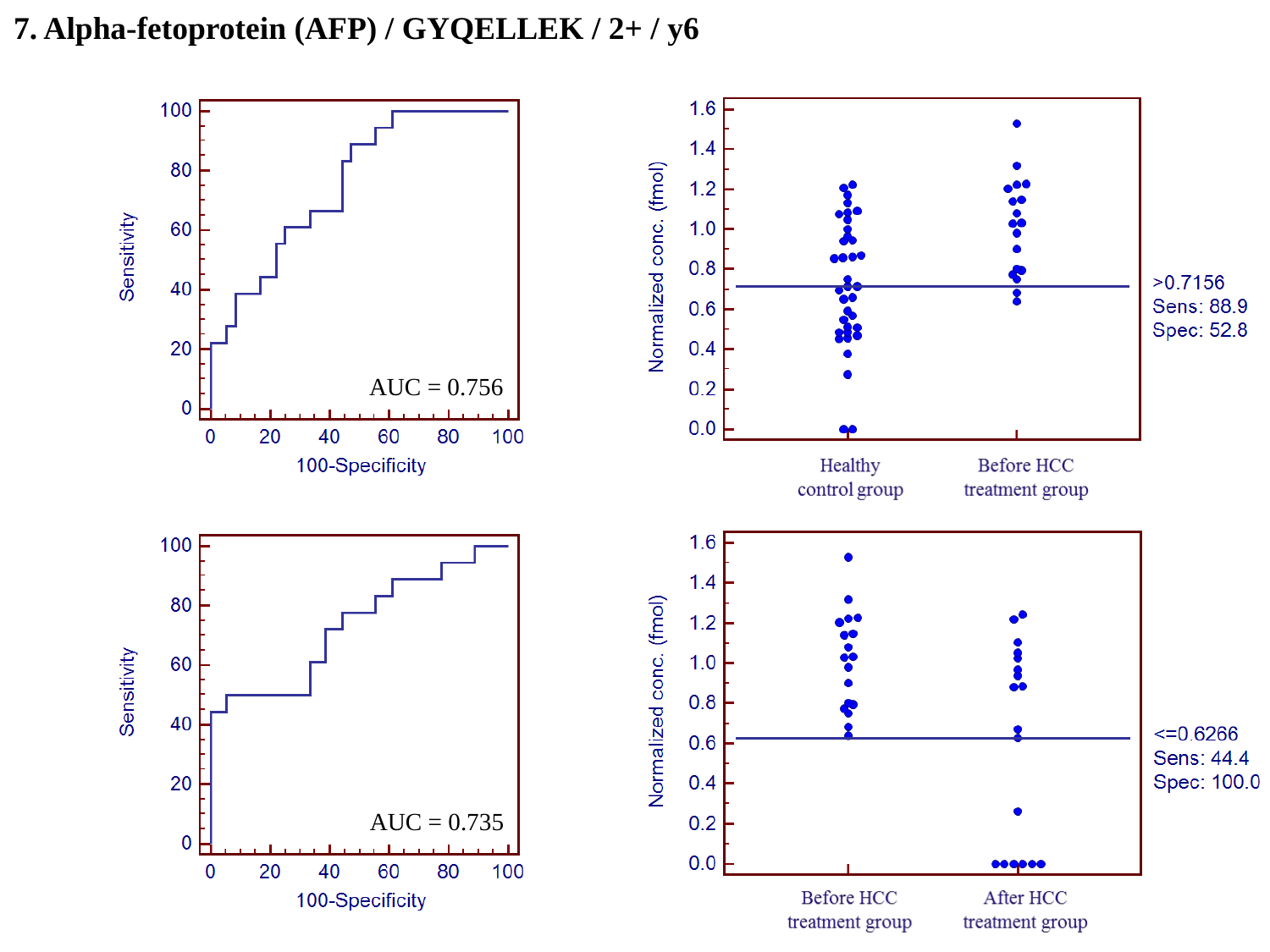

7. Alpha-fetoprotein (AFP) / GYQELLEK / 2+ / y6
AUC = 0.756
AUC = 0.735

## Slide 9
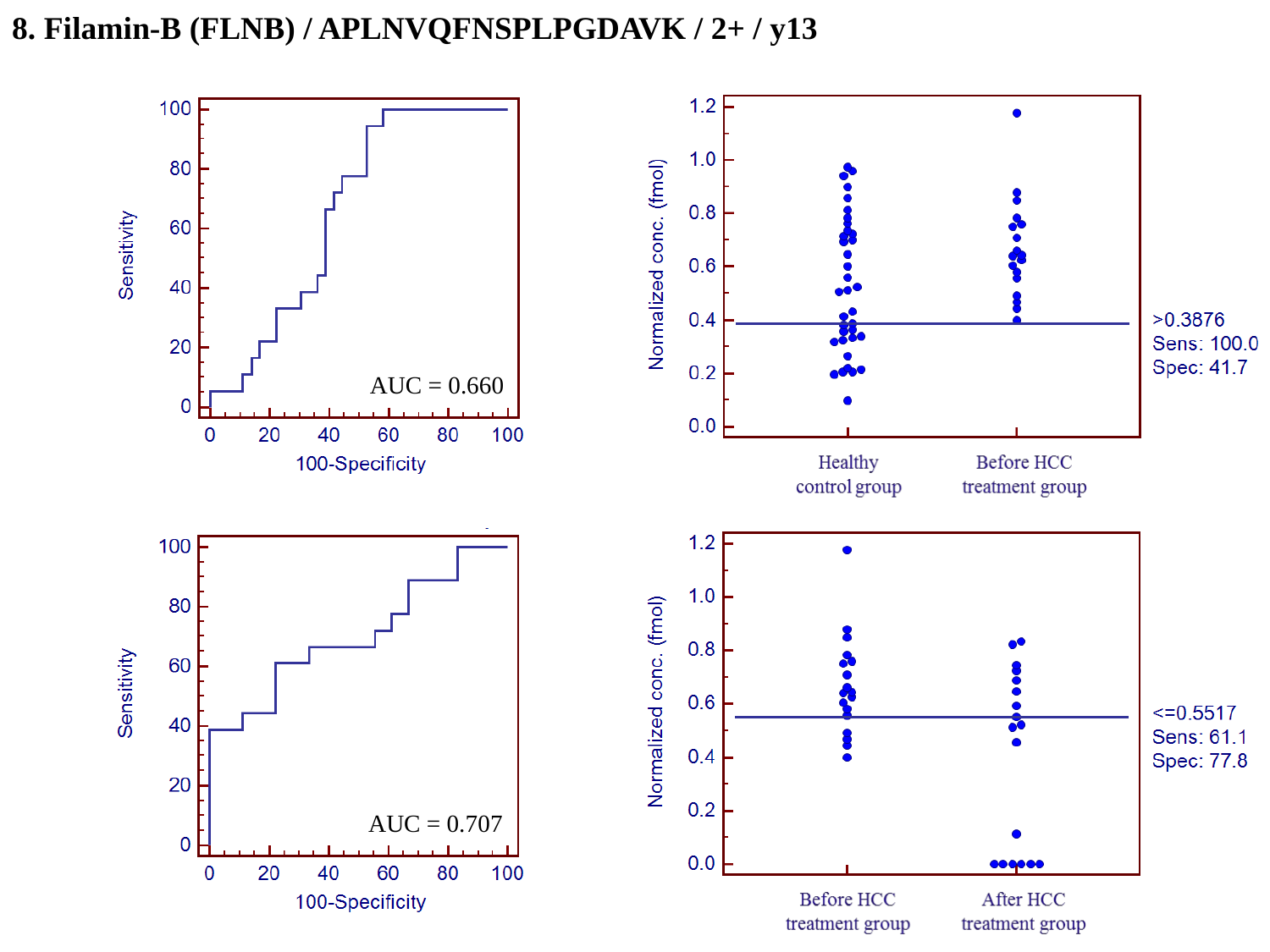

8. Filamin-B (FLNB) / APLNVQFNSPLPGDAVK / 2+ / y13
AUC = 0.660
AUC = 0.707

## Slide 10
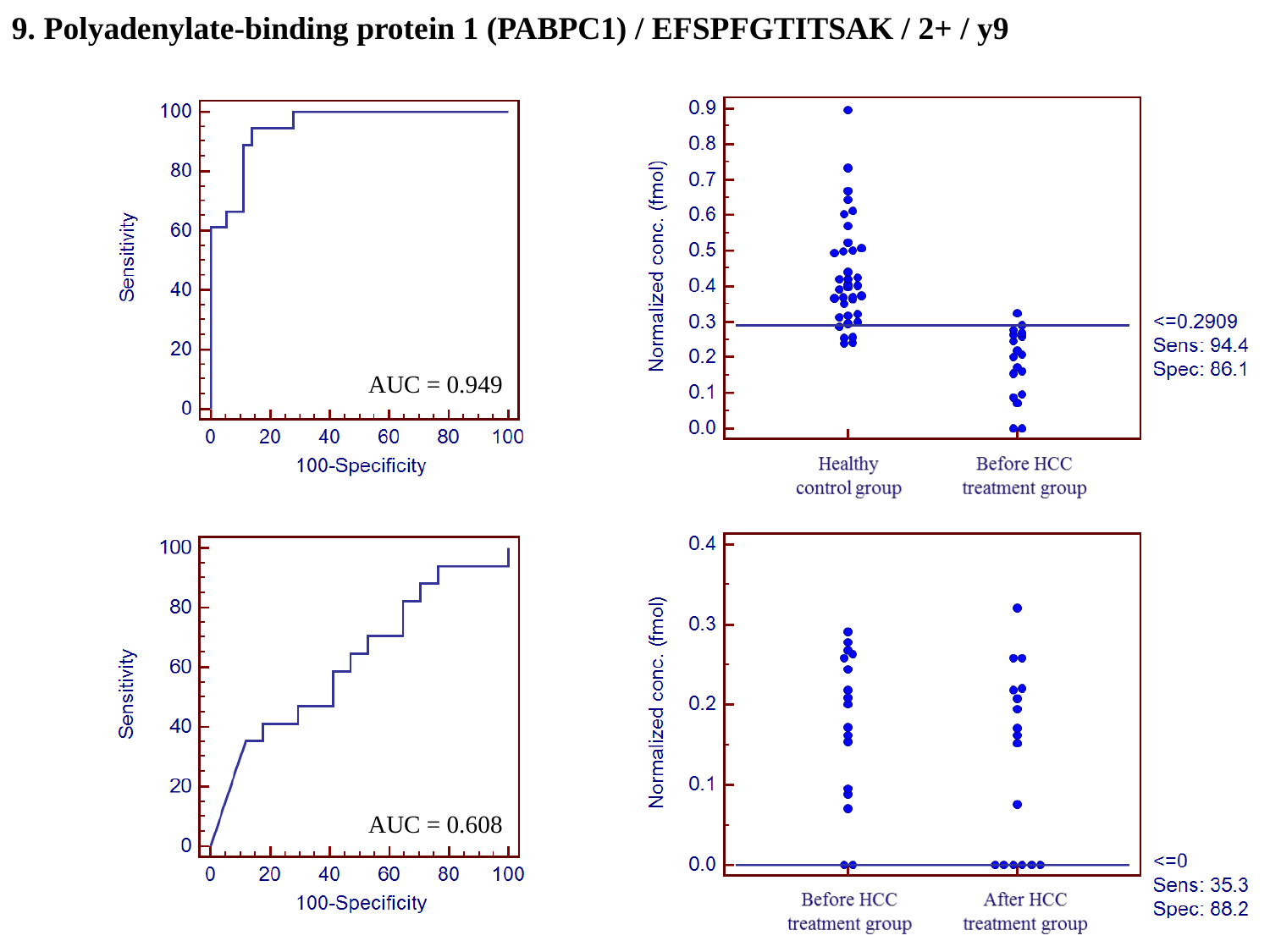

9. Polyadenylate-binding protein 1 (PABPC1) / EFSPFGTITSAK / 2+ / y9
AUC = 0.949
AUC = 0.608
